# Capacity-Building and Clinical Competence in Infectious Disease in Uganda: A Mixed-Design Study with Pre/Post and Cluster-Randomized Trial Components

**DOI:** 10.1371/journal.pone.0051319

**Published:** 2012-12-14

**Authors:** Marcia R. Weaver, Ian Crozier, Simon Eleku, Gyaviira Makanga, Lydia Mpanga Sebuyira, Janepher Nyakake, MaryLou Thompson, Kelly Willis

**Affiliations:** 1 Department of Global Health, University of Washington, Seattle, Washington, United States of America; 2 Accordia Global Health Foundation, Kampala, Uganda; 3 Mengo Hospital, Kampala, Uganda; 4 Mulago National Referral Hospital, Kampala, Uganda; 5 Infectious Diseases Institute, Makerere University, Kampala, Uganda; 6 Department of Biostatistics, University of Washington, Seattle, Washington, United States of America; 7 Accordia Global Health Foundation, Washington, D. C., United States of America; The George Washington University Medical Center, United States of America

## Abstract

**Trial Design:**

Best practices for training mid-level practitioners (MLPs) to improve global health-services are not well-characterized. Two hypotheses were: 1) Integrated Management of Infectious Disease (IMID) training would improve clinical competence as tested with a single arm, pre-post design, and 2) on-site support (OSS) would yield additional improvements as tested with a cluster-randomized trial.

**Methods:**

Thirty-six Ugandan health facilities (randomized 1∶1 to parallel OSS and control arms) enrolled two MLPs each. All MLPs participated in IMID (3-week core course, two 1-week boost sessions, distance learning). After the 3-week course, OSS-arm trainees participated in monthly OSS. Twelve written case scenarios tested clinical competencies in HIV/AIDS, tuberculosis, malaria, and other infectious diseases. Each participant completed different randomly-assigned blocks of four scenarios before IMID (t0), after 3-week course (t1), and after second boost course (t2, 24 weeks after t1). Scoring guides were harmonized with IMID content and Ugandan national policy. Score analyses used a linear mixed-effects model. The primary outcome measure was longitudinal change in scenario scores.

**Results:**

Scores were available for 856 scenarios. Mean correct scores at t0, t1, and t2 were 39.3%, 49.1%, and 49.6%, respectively. Mean score increases (95% CI, p-value) for t0–t1 (pre-post period) and t1–t2 (parallel-arm period) were 12.1 ((9.6, 14.6), p<0.001) and −0.6 ((−3.1, +1.9), p = 0.647) percent for OSS arm and 7.5 ((5.0, 10.0), p<0.001) and 1.6 ((−1.0, +4.1), p = 0.225) for control arm. The estimated mean difference in t1 to t2 score change, comparing arm A (participated in OSS) vs. arm B was −2.2 ((−5.8, +1.4), p = 0.237). From t0–t2, mean scores increased for all 12 scenarios.

**Conclusions:**

Clinical competence increased significantly after a 3-week core course; improvement persisted for 24 weeks. No additional impact of OSS was observed. Data on clinical practice, facility-level performance and health outcomes will complete assessment of overall impact of IMID and OSS.

**Trial Registration:**

ClinicalTrials.gov NCT01190540

## Introduction

Efforts to reduce the global burden of infectious disease are significantly constrained by shortages of trained health professionals and by deficits in quality of available care. [1]–[8] There is scant available evidence supporting different approaches to addressing these human-resource gaps. [9], [10]

Systematic reviews of capacity-building interventions report modest and significant improvements in clinical practice. A review of continuing medical education, [11] which included four studies in Africa, reported median improvements ranging from 6.9 to 13.6 percent. For systematic reviews of educational outreach visits [12] and audit and feedback, [13] which respectively included no studies and one study in Africa, the median improvements respectively ranged from 5.6 to 21 percent, and 5.0 to 16 percent. Focusing on low and middle income countries, Rowe et al. summarized “that the simple dissemination of written guidelines is often ineffective, that supervision and audit with feedback is generally effective, and that multifaceted interventions might be more effective than single interventions.” [10] Focusing on fever case management in Africa, Zurovac and Rowe reported that continuous quality improvement interventions were associated with better quality of care. [14] In their comparison of five studies with similar research design, in-service training improved the quality of care in one of five studies, and supervision visits in two of two studies.

In Uganda, in-service training for mid-level practitioners (MLPs) who are clinical officers and registered nurses, currently supports initiatives for the control of HIV/AIDS, [15], [16] childhood illness, [17] and malaria. [18] In this context, the Integrated Infectious Disease Capacity-Building Evaluation (IDCAP) undertook a prospective study of two different approaches to MLP training in infectious-disease care. The Integrated Management of Infectious Disease (IMID) training program included courses and distance learning. On-site support (OSS) was an educational outreach package of four activities over the course of two days per month at each health facility for nine months.

IDCAP combined a pre-post design for evaluation of the IMID training program with a cluster-randomized, trial for evaluation of OSS (randomized 1∶1 to parallel OSS and control arms). The IMID training program was chosen as the pre/post intervention, because a 3-week core course had become the standard for HIV/AIDS in-service training for doctors at Makerere University's Infectious Diseases Institute (IDI). [19]–[21] IMID tested extending it to MLPs, and covering a broader range of infectious diseases. OSS was chosen as the randomized intervention because there is little rigorous evidence on the effectiveness of educational outreach visits in Africa.

This paper addresses the effect of the IDCAP interventions on individual competence. Measures of individual clinical practice, facility-level performance, and health outcomes will be reported separately.

## Methods

The protocol for this trial and supporting CONSORT checklist are available as supporting information; see Protocol S1 and Checklist S1.

### Participants

**Figure 1:**
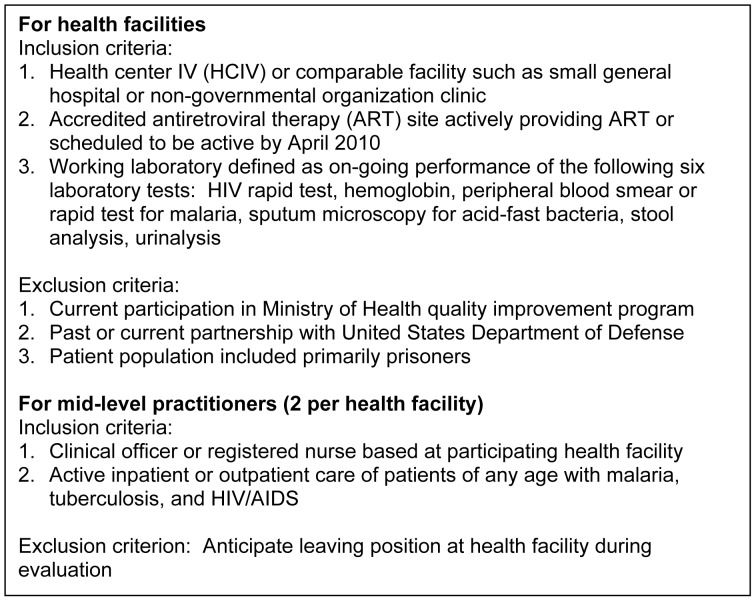
Inclusion and exclusion criteria for health facilities and mid-level practitioners.

Seventy-two MLPs were selected from 36 Ugandan health facilities. Inclusion and exclusion criteria for both health facilities and clinicians are in Figure 1. The 36 health facilities represented all administrative regions of Uganda.

Two MLPs were selected from each participating health facility. Registered midwives who met the inclusion criteria could be selected when a registered nurse was not available. All selected MLPs had a secondary school education; clinical officers had three years of pre-service training and two years of internship, registered nurses and registered midwives had three years of pre-service training, and registered nurse-midwives had four and one-half years of pre-service training. Preference was given to eligible MLPs with two characteristics: 1) held leadership roles such as in-charge of ward or clinic, or were focal person for malaria, TB, HIV, or prevention of mother-to-child transmission of HIV (PMTCT); and 2) had previous training and experience in counseling and/or Integrated Management of Childhood Illness (IMCI), Integrated Management of Adolescent and Adult Illness (IMAI).

### Ethics Statement

IDCAP was reviewed and approved by the School of Medicine Research and Ethics Committee of Makerere University (reference number 2009-175) and the Uganda National Committee on Science and Technology (reference number HS-722). Written informed consent was obtained from participants for secondary analysis of IDI training program data. The University of Washington Human Subjects Division determined that it did not meet the regulatory definition of research under 45 CFR 46.102(d).

### Interventions

**Figure 2:**
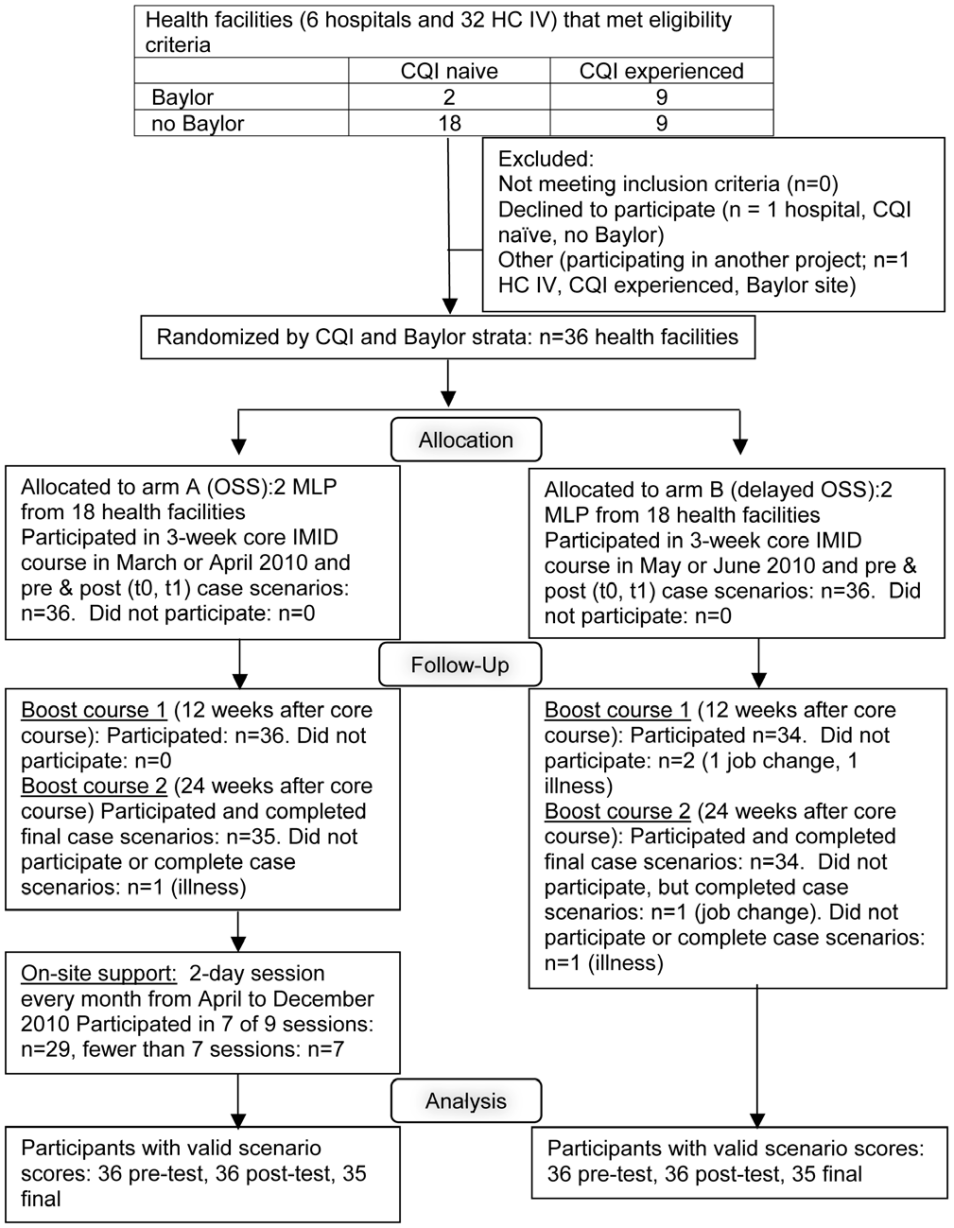
Flow diagram of mid-level practitioners who attended the Integrated Management of Infectious Diseases course. The figure shows the selection and random allocation of health facilities to two arms. Participants in arm A attended the Integrated Management of Infectious Disease (IMID) training program and On-Site Support. Participants in arm B attended IMID.

Two interventions sought to support the development of routine and complex clinical reasoning skills: IMID training program and OSS. As shown in Figure 2, all 72 MLPs participated in IMID core training from March to June 2010. Thirty-six MLPs at 18 randomly selected facilities participated in OSS beginning in April 2010 after the first session of the IMID core course. The interventions and the evidence in the medical education literature supporting them are described in detail in Miceli A, et al. [22]

The IMID training program began with a 3-week core course, followed over a 24-week period by two 1-week boost courses and distance learning. In 2010, a 3-week session was offered to participants in arm A in either March (A1) or April (A2) 2010, and to arm B in May (B1) or June (B2). Twelve and 24 weeks after the 3-week IMID course, all participants participated in 1-week boost courses. The start and end dates were different for each session, but the duration of follow-up was the same. The second boost course for the A1 session finished on 1 October 2010 and for the B2 session on 17 December 2010.

The 3-week IMID core course addressed diagnosis and management of HIV/AIDS, malaria, TB, diarrhea, acute respiratory infections and other infections of local importance in pregnant women, non-pregnant adults, infants and children. Its content was specifically adapted to Ugandan national policy and to the clinical context of a health center IV (HC IV), which included responsibility for child health and prevention, management, and control of infectious diseases within a health subdistrict. [23] The content was summarized in 14 Clinical Decision Guides. The core course and boost courses included both classroom sessions taught at IDI by expert clinicians in Kampala, Uganda and12 half-day clinical rotations. Distance learning between courses used a case-based method; participants reflected on and recorded cases encountered in their home health facilities using structured log books. Review of original IMID content during boost courses built on the participants' cases from the log books.

All participants were also encouraged to use the AIDS Treatment Information Center (ATIC), which is a Kampala-based warm-line staffed by medical doctors and pharmacists experienced in infectious disease, for advice on management of complicated patients.

The facilities randomized to Arm A (1∶1 allocation) participated in monthly OSS beginning in April 2010. Arm B participated in OSS beginning in March 2011, but the impact of OSS on clinical competence in Arm B was not assessed. OSS was provided by a four-member mobile team: medical doctor, clinical officer, registered nurse and laboratory technologist. The teams' two-day visits included multidisciplinary didactic sessions, discipline-specific break-out sessions, mentoring for both clinical and laboratory staff, and continuous quality improvement (CQI) activities. Each OSS visit was structured around a theme, beginning with “Emergency, Assessment, Triage, and Treatment.”

The OSS sessions for clinicians were based on IMID core course materials. The multi-disciplinary training was primarily an overview of national guidelines. The IMID participants attended the break-out session for clinicians, which focused on the Clinical Decision Guides. For the mentoring sessions for clinicians, all of the mentors attended the pilot version of the IMID core course. The mentoring sessions varied however, with the patients at the facility during the OSS visit. The mentors sought to build the following six patient care competencies: 1) history taking, 2) routine physical examination including danger signs, 3) clinical reasoning including identification of differential diagnoses, 4) ordering laboratory investigations, 5) appropriate diagnosis, and 6) appropriate treatment and management plan.

The CQI activities were designed to support the CQI teams at the sites, which included IMID participants, and focused on a subset of 13 of the facility performance indicators. All of the indicators were selected in collaboration with the curriculum developers for the IMID core course and reflected its content. Some indicators measured individual clinicians' performance, such as reducing the percentage of patients with a negative malaria smear who were treated with anti-malarials. Others measured team performance, such as increasing the percentage of malaria suspects with laboratory tests for malaria for which the clinician ordered the test and the laboratory staff performed it. The sites generally chose to focus on six indicators. Three CQI activities for each visit were organized around those indicators: 1) preparing data on the indicators, 2) mapping processes of care, and 3) reviewing the data and processes of care to identify problems and goals for the next month.

### Objectives

The hypothesis for the single-arm intervention with pre-post design was that the IMID course is effective at building clinical competence, where competence was measured by participating clinicians' scores on written case scenarios. The hypothesis for the cluster-randomized trial component was that OSS will yield additional improvement in the competence of individual MLPs relative to those in the control arm, using the same measure.

The primary objectives of our assessment of the impact of IDCAP interventions on individual clinician competence were:

1. Estimate mean change in written case scenario scores from t0 (baseline) to t1 (end of 3-week IMID course) for arms A and B combined. (Between t0 and t1, both arms received the same intervention.)
2. Compare mean changes in written case scenario scores from t1 to t2 (end of second boost course) between arm A and arm B. (Between t1 and t2, only arm A received OSS.)
3. Estimate overall mean changes in scenario score for arms A and B from t0 to t2 and t1 to t2.

Secondary objectives were description of association of scores with characteristics of scenario administration, and differences in scores over testing points for individual case scenarios.

### Outcomes

The primary outcome was change in aggregate scores (all participants or across arms, depending on objective) on written case scenarios across three time intervals (t0–t1, t0–t2, t1–t2). For the cluster-randomized trial only, the primary outcome was the difference in score change across study arms from t1 to t2.

We selected written case scenario scores, sometimes referred to as vignettes, as our primary measure of competence, because case scenarios test both knowledge and clinical reasoning skills. Case scenario scores have been validated as a measure of quality of care against data from clinician encounters with standardized patients in the United States. [24] Case scenario scores have also been used successfully to describe differences in clinician competence across groups characterized by practice setting, level of training, and other factors. [25]–[29] Recently, Das et al. identified a gap between case scenarios and standardized patients or observation of clinical care as measures of practice, called the “know-do-gap,” among doctors in India and Tanzania. [30] Leonard et al. noted differences in the gap across professions in Tanzania, but the differences were associated with the organization where they practiced rather than years of training. [31]

Twelve case scenarios were designed to cover the main elements of IMID content. A sample case scenario is presented in Web Appendix S1. Scenario structure was based on a template that included danger/emergency signs, history, physical examination, laboratory testing, initial diagnosis and treatment, and evolution of the case over time (hours to months).The template document also referenced specific IMID curriculum sessions and Ugandan national policy documents that addressed the subject of each question. Scenario questions were short-answer and open-ended (e.g. “What are the three most likely causes of this patient's current signs and symptoms?”). In a pre-trial pilot, scores on draft versions of the case scenarios increased significantly after MLP exposure to a pilot version of the IMID core course (Weaver M et al., unpublished manuscript).

Available time for assessment did not allow for administration of all 12 case scenarios to each participant at a single testing point. In addition, each case scenario was structured in four parts where the answers to one part were revealed at the beginning of the next part (except the fourth). To isolate course learning as opposed to familiarity with the case scenarios, participants responded to different case scenarios at each testing point. Consequently, the 12 case scenarios were divided into three blocks of four scenarios, and each block was assigned to participants from one-third of the sites within each arm at each testing point. Each block contained material relevant to HIV/AIDS, malaria, tuberculosis, and selected other infectious diseases in pregnant women, non-pregnant adults, and infants/children. The competencies differed across blocks; for example case scenario 2 in block A addressed AIDS in a non-pregnant adult, whereas case scenario 10 in block C addressed AIDS in a pregnant woman. Table 1 briefly describes the content of the case scenarios and their distribution across blocks.

**Table 1:** Scenario Content Description; Evolution of Scores on New Scenarios by Scenario and Block.

	Patient subgroup	Presenting complaint(s)	Disease entities	Mean score, t0 (%correct)	Mean score, t1 (% correct)	Mean score, t2 (% correct)
	**Block A**			**40.1**	**50.5**	**51.1**
1	Non-pregnant adult	Cough, fever, vomiting, abdominal pain	TB (smear positive), adverse drug reaction (severe)	59.1	66.3	65.9
2	Non-pregnant adult	Sore throat, dysphagia, fever, chest pain	AIDS, esophageal candidiasis,TB (suspected extrapulmonary)	32.8	46.0	43.8
4	Infant	Coma, fever, vomiting, perinatal HIV exposure	Bacterial meningitis, HIV exposure, PMTCT*	34.9	39.8	46.6
12	Child	Cough, fever, diarrhea, wasting	Pneumonia, adverse drug reaction (minor), PMTCT*	33.8	50.0	48.0
	**Block B**			**41.5**	**48.8**	**50.6**
5	Child	Fever, respiratory distress, wasting	Malaria (severe), pneumonia, severe acute malnutrition, anemia	38.2	49.6	50.3
6	Pregnant woman	Fatigue, post-partum fever	Malaria (uncomplicated), post-partum endometritis, anemia	55.1	61.0	64.0
9	Infant	Clinical worsening on ART	AIDS treatment failure, suspected HIV encephalopathy	31.4	34.5	38.1
11	Non-pregnant adult	Diarrhea (recurrent), weight loss	Diarrhea (cholera), HIV/AIDS (wasting syndrome)	41.3	50.0	50.1
	**Block C**			**36.3**	**48.1**	**47.1**
3	Non-pregnant adult	Fever, convulsions	Malaria (severe), HIV infection	34.0	46.9	43.3
7	Infant	Diarrhea, lethargy	Neonatal sepsis	37.1	47.9	46.0
8	Non-pregnant adult	Adenopathy,clinical worsening on ART	TB-IRIS, adverse drug reaction (severe)	36.5	51.5	55.6
10	Pregnant woman	Cough, fever	AIDS, smear negative pulmonary TB	37.5	45.9	43.4

Abbreviations. AIDS: acquired immune deficiency syndrome; ART: antiretroviral therapy; HIV: human immunodeficiency virus; IRIS: immune reconstitution inflammatory syndrome; PMCT: prevention of mother to child transmission (of HIV); TB: tuberculosis.

* Although they did not focus on pregnancy, these scenarios also addressed antenatal and post-partum PMTCT protocols.

**Figure 3:**
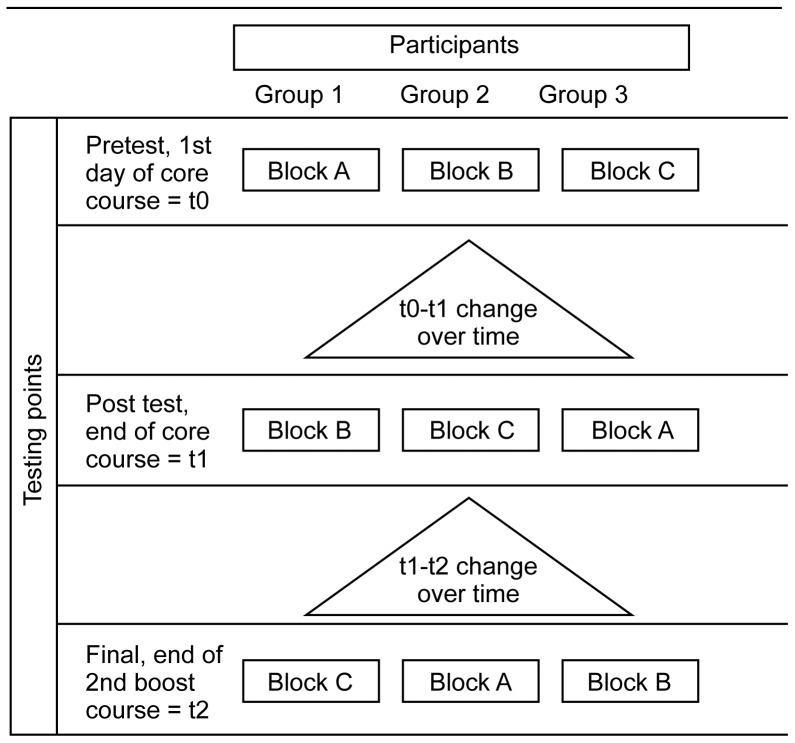
Allocation of case scenarios across testing points. The 72 IDCAP participants (36 from arm A, 36 from arm B) were randomly assigned to three groups, which contained 12 participants from each arm. The 12 clinical case scenarios were distributed across three, 4-scenario blocks (A, B, and C), and each group was assigned to a different sequence of blocks.

Within each arm, at t0 12 participants (two from each of six randomly selected sites) completed block A, participants from another six sites completed block B, and participants from the remaining six sites completed block C. As shown in Figure 3, the block allocations were then rotated at subsequent testing points, so that participants completed different blocks at t1 and at t2. Within each arm, all 12 scenarios were completed by 12 participants each time; over the three testing points, each participant completed all 12 scenarios. This design allowed us to compare mean scenario scores across arms and time points, but not evolution of scores at the level of the individual participant.

To mitigate against the possible impact of fatigue or time constraints on case scenarios scores, the order of the scenarios within each block was also randomized for each participant. Within each block of four case scenarios, there were 24 (4!) possible sequences; for example 1234 and 1243. The sequence for the first block was repeated in subsequent blocks; for example a participant whose sequence in block A at t0 was 1234, had sequence 5678 in block B at t1. We selected 12 of 24 possible sequences for each block and randomly assigned one sequence to each of the 12 participants assigned to that block in each arm. The same 12 sequences were assigned to each arm, so the sequences were balanced across arms.

To test whether score improvements on repeated case scenarios reflected course learning or familiarity with case scenario content, each participant repeated one (at t1) or two (at t2) randomly selected scenarios from earlier testing points. Their position in the sequence for the current time was also selected at random. Pretest (t0) case scenarios that were selected to be repeated at post test (t1) were removed from possible selection for final test (t2).

Secondary outcomes were differences in scores on new case scenarios (scenarios not previously seen by the individual participant) and repeat scenarios (scenarios that were completed by the same participant at more than one time), differences in scores associated with the order in which case scenarios were completed, and differences in scores associated with individual scenarios.

Two experienced Ugandan physicians scored the scenarios based on pre-specified scoring guides developed specifically to reflect IMID training program content. To eliminate inter-scorer variability, a case scenario was always scored by the same person (Weaver M, et al., unpublished manuscript). After t1, the four co-authors who are clinicians (IC, SE, MG, JN) and Paula Brentlinger reviewed the participants' answers at t0 and t1 to identify correct answers that had not been anticipated in the original scoring guidelines; for example, clinical actions that were technically correct but generally not relevant in the HC IV context, such as requesting computerized tomography of the brain. They also reviewed changes in Ugandan national policy guidelines and/or IMID training program content that had occurred after the case scenarios were drafted. The scoring guidelines were revised and expanded, based on consensus. The revised guidelines were used to score all of the scenarios and those scores are reported below.

### Sample size

The sample size calculations for IDCAP were based on testing the effect of OSS on facility performance, and thus were not based on power requirements for the analysis of the case scenarios. The sample size calculations are reported in Naikoba S. et al. (unpublished manuscript).

Each facility selected two MLPs to attend the IMID training program for a total of 36 MLPs per arm. The initial proposal was to train all the MLPs at the site, based on evidence of the effect of IDI's 3-week Comprehensive HIV Care Including ART course for doctors [21] and 1-week Integrated Management of Malaria course for teams of MLPs, laboratory professionals, and records staff [18] on clinical practice. Funding was not available to train all MLPs at 36 sites however, so the effectiveness of IMID for two MLPs per facility was tested in the hopes of offering it to all MLPs in the future.

### Randomization—Sequence generation

Health facilities were assigned to arm A (OSS) or arm B (delayed OSS) by stratified random selection (see Figure 2). Sites were stratified by two characteristics: 1) prior experience with the Health Care Improvement project, a CQI program for HIV prevention and treatment vs. CQI naïve, and 2) current or prior support from the Baylor International Pediatric AIDS Initiative (BIPAI) for clinical mentoring in pediatric HIV/AIDS vs. no BIPAI (for more information, please see http://www.bipai.org/Uganda/). Sites were then randomly assigned to arm A or B (1∶1 balance) within those strata.

Randomization of health facilities to arm was implemented using random number generation in Stata 10.1. As noted above, sequences of case scenarios were also randomly assigned to participants so that the case scenarios sequences would be balanced across arms at all three time points, also using the same method.

### Randomization—Allocation concealment

Randomization to arm A or B occurred on February 23, 2010 after the majority of participants had completed baseline clinical assessments to measure clinical performance in January and February 2010. Within two weeks of the A1 session of the IMID core course, arm A participants were notified of their upcoming course dates and arm assignment. Allocation was not concealed during the IMID training program and testing points. Randomization of participants to sequences of case scenarios occurred on March 17, 2010 before the A1 session of IMID.

### Randomization—Implementation

The generation of random sequences was performed by the co-author who is a biostatistician (MLT) and who was not involved in site selection or participant enrollment. Participants were assigned to interventions based on the allocation of their home health facility to arm.

### Blinding

This study was not blinded.

### Statistical methods

For estimation of mean aggregate score changes for the three possible time intervals (t0–t1, t1–t2, t0–t2), we used linear mixed-effects models with individual score on a single new case scenario as the dependent variable, time and scenario as fixed effects, and participants nested within health facility as random effects. The inclusion of a random effect for health facility did not meaningfully alter the results and this variable was not included in the analyses reported below. For assessment of score differences across arms between t1 and t2 (the only interval in which the two arms received different interventions), we included an interaction between time and arm.

For comparison of new vs. repeat scores, we used the model described above with individual scores on all case scenarios, with the addition of a dichotomous variable for new vs. repeat scenario. We assessed the effect of scenario order using the model described above and all case scenarios, including a categorical variable for order in which the scenario was completed and, alternatively, including linear spline terms.

We conducted exploratory analyses with a model that included dummy variables for hospital, registered nurse, and registered midwives to control for their effects, because facility-type and profession of the participants were not balanced across arms (see baseline data below).

All analyses were conducted in Stata 11.0 (StataCorp LP, College Station TX, 2009). The probability of type I error to define statistical significance was 0.05 and all tests were two-sided.

## Results

### Participant flow

Figure 2 describes participant flow. Sixty-eight of 72 participants attended all three IMID courses and completed case scenarios on each testing point. Two participants missed boost course 1, and three missed boost course 2. One of those who missed boost course 2 completed the case scenarios at t2. One participant who missed the 1^st^ boost course while on study leave attended the second boost course and completed the scenarios at t2. Twenty-nine participants attended at least seven of nine monthly OSS sessions.

### Recruitment

Recruitment of health facilities occurred between March and September 2009. Identification of participants occurred between June 2009 and February 2010. Participants included 46 (64%) clinical officers, and 22 (30%) registered nurses, and four (6%) registered midwives. Thirty-seven (53%) of the participants were men. For assessment of evolution of clinical competence, the follow-up period extended from the first day of the 3-week core course to the last day of boost course 2.

### Baseline data

After stratified random assignment, eight (44%) and nine (50%) of 18 health facilities in arms A and B, respectively, were HCI experienced, and five (28%) in both arms had participated in BIPAI. The distribution of hospitals and clinical officers was not balanced across arms. One (6%) and four (22%) of the health facilities in arms A and B, respectively were hospitals. Among participants, 24 (67%) and 22 (61%) in arms A and B, respectively, were clinical officers. All four registered midwives were in arm B.

### Numbers analyzed

Complete scenario scores were available for all 72 participants at both t0 and t1. At t2, two participants (one from each arm) were not present, and no scores were available for them. Two participants (both from the control arm) did not attend all three courses, but agreed to complete the scenarios at the end of the 2^nd^ boost course so that their scores were included in an “intention-to-treat” analysis. Thus, scores were available for 856 new scenarios (12 each for 70 of 72 participants; eight each for two participants who were absent for the second boost course).

In addition to the new scenarios, we had scores for 236 repeated scenarios, administered at t1 and t2. The anticipated number of repeat scenarios was 216. The two participants who did not attend the second boost course accounted for four missing repeat scenarios. An error in scenario administration for the A2 session at t1 resulted in the unintended administration of 24 excess repeat scenarios (1 or 2 per person, for a total of six or seven scenarios at t1, instead of the anticipated five). We included all 236 repeat scenarios in these analyses.

### Outcomes and estimation

For the pre/post period (t0 to t1), in which the intervention did not differ across arms, we observed significant scenario score increases for both arms (Table 2). The aggregate mean score change (95% confidence interval, p-value) from t0 to t1 was +9.8 ((8.0, 11.6), p<0.001).

**Table 2:** Aggregate Mean New Scenario Scores and Score Changes by Arm and Time.

	Percentage of Points Correct			Absolute Change in Percentage of Points Correct		
	(95% CI)			(95% CI), p-value		
	t0	t1	t2	t0–t1	t1–t2	t0–t2
OSS arm	37.4	49.5	49.0	+12.1	−0.6	+11.5
	(34.3,40.6)	(46.4,52.7)	(45.8,52.2)	(9.6,14.6), p<0.001	(−3.1,1.9), p = 0.647	(9.0,14.1), p<0.001
Control arm	41.2	48.7	50.2	+7.5	+1.6	+9.1
	(38.0,44.4)	(45.5,51.8)	(47.0,53.4)	(5.0,10.0), p<0.001	(−1.0,4.1), p = 0.225	(6.5,11.6), p<0.001
Both arms	39.3	49.1	49.6	+9.8	+0.5	+10.3
combined	(37.1,41.5)	(46.9,51.3)	(47.4,51.9)	(8.0,11.6), p<0.001	(−1.3,2.3), p = 0.594	(8.5,12.1), p<0.001

Abbreviations. t0: Pretest (baseline); t1: End of 3-week core course; t2: End of 2^nd^ boost course (24 weeks after t1).

For the randomized parallel-arm period (t1 to t2) in which arm A received OSS but arm B did not, mean scores remained essentially stable for both arms, and there was no statistically significant difference in mean score change over time across arms (Table 2). The estimated mean difference in t1 to t2 score change for arm A vs. arm B was −2.2 ((−5.8, 1.4), p = 0.237). Combining both arms, the mean score change for t1–t2 was +0.5 ((−1.3, 2.3), p = 0.594).

Similarly, the difference in mean score change from t0 to t2 was not statistically significant across arms; the increase was 2.5 points higher ((−1.1, 6.1), p = 0.179) for arm A. Combining both groups, the mean score change from t0 to t2 was +10.3 ((8.5, 12.1), p<0.001).

Although absolute scores differed substantially by scenario, the pattern of score change over time was quite similar across all 12 scenarios (Table 1). For each time period, mean scores were similar for the three blocks, but patterns of change for each block varied across time. The t0 to t1 increase varied from 7.3 to 11.8 percent, and the t1 to t2 change from −1.0 to +1.8 points, across blocks.

Our analysis of secondary outcomes revealed small but statistically significant differences between new and repeat scenario scores, and statistically significant associations of score with scenario order. At t1 and t2, mean scores on new scenarios were, on average, 1.9 points lower ((−3.6, −0.2), p = 0.026) than scores for repeated scenarios. In analysis that combined all three time points, scores on the fourth, fifth and sixth case scenarios were statistically significantly lower than the first case scenario. In analyses with a linear spline with a knot at scenario order 4, order had a slope of −0.5 ((−1.2, 0.1), p = 0.103) (change in score percent per one unit increase in order) for order ≤4 and −3.0 ((−4.2, −1.8), p<0.001) for order ≥4.

### Ancillary analyses

We conducted sensitivity analyses to control for facility type and profession of the participants, because there were more hospitals and midwives in arm B than A. The mean score changes were almost identical to those reported in Table 2, and the estimated mean difference in t1 to t2 score changes for arm A vs. arm B was −2.2 ((−3.4, 7.7), p = 0.448).

We also conducted an exploratory analysis that omitted the scenario results of participants who missed either or both of the boost courses. In this analysis (n = 68), the estimated mean difference in t1 to t2 score change for arm A vs. arm B was 1.1 ((−4.5, 6.8), p = 0.693) points, consistent with the results reported above.

### Adverse events

One participant was injured in a motor vehicle accident while traveling to Kampala to attend the second boost course, which was within the scope of her employment by the Ministry of Health. No other adverse events or side effects were reported for either arm.

## Discussion

### Interpretation

The IMID training program resulted in statistically significant increases in the clinical competence of participants between pretest and after the 3-week core course, and these improvements were maintained, for participants in both arms, through the subsequent 24 week training program. These increases were measured with scores of new cases scenarios. Scores on repeat scenarios were statistically significantly higher than new scenarios, reflecting the small effect of familiarity with the scenarios' content. IMID training increased competency in domains relevant to multiple different infectious diseases and patient subgroups, because score increases were demonstrated for all 12 scenarios and for all three blocks.

Between t1 and t2, there was no significant difference in score change across arms. The effect size was small, indicating that absence of statistical significance was not a consequence of inadequate sample size. Although this suggests that OSS did not significantly add to the development of clinician competency, it is also possible that OSS reinforced competencies that were not well-represented in the case scenarios. Although the OSS training materials were based on IMID training materials, the case scenarios were designed before the OSS training materials, and consequently without reference to them. Improvements in clinical practice and facility performance would be reflected in those measures.

Concerning the mechanics of scenario administration, order of scenario administration is important; statistically significantly lower scores for four or more suggested that participant fatigue may influence scores. If scenario order is not randomized, it should be the same for all participants for on-going courses or across arms for comparison.

Also, it was difficult to tailor different scenarios to test the same competencies. Comparisons of individuals who completed different blocks at each testing point were not possible; someone who completed block A with the lowest average scores at t0 and block C with the highest average scores at t1 might show an increase on average, whereas those who completed the opposite might show a decrease. Given the modest increase associated with the repeat case scenarios, using the same scenarios at two or more time points would be more comparable than using different scenarios addressing similar competencies.

### Limitations

Scenario scores are well-validated measures of competence, but may overestimate clinical performance. [30] For this reason, IDCAP has also assessed individual practice and facility-level clinical performance, and will report them separately. Scenarios completed by participants with poor handwriting or less advanced English-language skills may have been underscored. The intentionally challenging content of the scenarios may have discouraged some participants. Finally, the research design did not allow comparison of competence across professions or types of facilities.

### Generalizability

Concerning facilities, the primary results may generalize to other settings without an on-going CQI program and with de facto task shifting. Uganda's National AIDS Control Program benefited from two large scale, on-going CQI programs, which would have confounded the measurement of the effects of OSS on HIV care. Hence IDCAP's eligibility criteria focused on a narrow range of health facilities to isolate the effect of OSS. Consequently, the effects on competence in HIV care may not generalize to other facilities that are accredited to provide ART within Uganda. They may however, generalize to facilities that serve patient populations at risk for malaria, tuberculosis and other common infectious diseases within Uganda, and to other contexts in which CQI interventions for HIV care were not on-going.

With regard to clinicians, the primary results may generalize to other active clinicians with leadership roles and previous training in counseling, IMCI or IMAI. IMID participants were not chosen at random, and these results would not necessarily generalize to junior clinicians or those with less in-service training. Evidence from the Integrated Management of Malaria course [18] however, suggested that all MLPs would potentially benefit.

The IMID training program used material developed and adapted specifically to Ugandan clinical officers and registered nurses and to the local policy and clinical environment, with real-time updates to reflect changing local Ugandan policy. As with other training initiatives such as IMCI and IMAI, the content of the IMID curriculum materials and case scenarios would require adaptation before use in other countries or for other cadres, [32] and the magnitude of observed evolution of scenario scores might vary in other contexts.

The secondary results may also generalize to other applications of case scenarios to test the effect of training on clinical competence. Our findings that scenario scores varied significantly depending on new vs. repeat status and on order, and that scores differed across scenarios that addressed similar infectious-disease problems in different ways, are likely to be applicable to other similar evaluations of capacity-building interventions.

### Overall evidence

In spite of recent international emphasis on programs that encompass more than one disease entity and more than one target population, only a handful of integrated programs have been implemented and evaluated. [33]–[35] IDCAP's interventions were an innovative approach to supporting clinicians in development of routine and adaptive reasoning skills. Clinical competence was measured by case scenarios, which was the first application of this measure for longitudinal evaluation of a capacity-building program. The case scenarios and scoring guidelines carefully reflected the IMID training program's content. In addition to randomized allocation of health facilities to parallel arms assigned to OSS or delayed OSS, the novel research design exploited randomization to permit more extensive assessment of curriculum content than would otherwise have been possible, and to test different approaches to scenario administration. The IMID core course was effective at improving clinical competence. Although the measure of competence failed to demonstrate an additional impact of OSS, forthcoming data on the impact of OSS on measures of individual clinical practice, health facility performance, and health outcomes suggested that that both IMID and OSS may have contributed to improving the quality of care.

## Supporting Information

Appendix S1 **Web appendix.**
(DOCX)

Protocol S1 **Trial Protocol.**
(DOC)

Checklist S1 **CONSORT Checklist.**
(DOC)
