# Supplementary material for: Capacity-Building and Clinical Competence in Infectious Disease in Uganda: A Mixed-Design Study with Pre/Post and Cluster-Randomized Trial Components
Source: PLoS One. 2012 Dec 14;7(12):e51319. doi: 10.1371/journal.pone.0051319 (PMC3522731; doi:10.1371/journal.pone.0051319)
Supplement: Appendix S1 — Web appendix. (DOCX) [file pone.0051319.s001.docx]

***IDCAP***

***Scenario 1***

***Part 1***

***THIS SCENARIO HAS 4 PARTS AND 16 QUESTIONS.***

**PART 1 (OF 4).**

************************************************************************************

***Case description:*** Lucas is a 25-year-old man with cough.

***Quick check for danger signs:***  No danger signs.

***Focused history:***  Cough x 1/12, with night sweats and fatigue. No hemoptysis or weight loss. Previously healthy; never had any serious illness of any kind. No prior treatment for the present illness except for paracetamol. Never tested for HIV, but has multiple current sexual partners.

***Focused physical exam:***  T 38.1 axillary, RR 18, Pulse 90/regular, BP 112/74, BMI 18.4, weight 65 kg. General: No abnormal findings. Neuro: Awake and alert, no deficits. Head, eyes, ears, nose, throat: No abnormal findings. Lungs: inspiratory and expiratory rhonchi over the left posterior lung field. Cardiac and abdominal exams: No abnormal findings.

***Laboratory investigation:*** 2 of 2 sputum smears positive (++) for AFB. HIV antibody testing negative. A malaria slide shows no *P falciparum*.

**********************************************************************************

| **Question 1. Question 1.** What is Lucas’s diagnosis? (Name 1 disease.) | For scorers only. | |
| --- | --- | --- |
| **Answer to question 1: ______________________________________________** |  |  |

| **Question 2. How would you CLASSIFY this case*?*** | For scorers only. | |
| --- | --- | --- |
| **Answer to question 2: ___________________________________________** |  |  |

| **Question 3. What treatment CATEGORY does Lucas fall in*?*** | For scorers only. | |
| --- | --- | --- |
| **Answer to question 3: _______________________________________________** |  |  |

| **Question 4. What medications should Lucas start taking today, or as soon as possible? You may list up to 6 medications.** | For scorers only. | |
| --- | --- | --- |
| **Answer to question 4:**   1. __________________________________________________ 2. __________________________________________________ 3. __________________________________________________ 4. __________________________________________________ 5. __________________________________________________ 6. __________________________________________________ |  |  |

| **Question 5. If given the treatment described above, what signs and symptoms of adverse drug reaction should Lucas be warned about before starting treatment? List 3 signs or symptoms of minor side effects and 3 signs or symptoms of major side effects.** | For scorers only. | |
| --- | --- | --- |
| **Answer to question 5: Possible signs or symptoms of minor side effects:**   1. **_______________________________________________________** 2. **_______________________________________________________** 3. **_______________________________________________________**   **Possible signs or symptoms of major side effects:**   1. **__________________________________________________________** 2. **__________________________________________________________** 3. **__________________________________________________________** |  |  |

| **Question 6. What are the most important follow-up tests that Lucas will need (at future visits), and when should they be done? Name 2.** | For scorers only. | |
| --- | --- | --- |
| **Answer to question 6:**   1. **______________________ (test) should be done in _________ (time).** 2. **______________________ (test) should be done in _________ (time)** |  |  |

**End of part 1*.***

***Please fold this part of the case and place it in the envelope. Then open the binder and remove the next part of the case, and close the binder. Remember: You may have only one section of the case open at a time. You cannot go back and look at earlier sections of the case once you have placed them in the envelope.***

***IDCAP***

***Scenario 1***

***Part 2***

**Beginning of part 2 (OF 4 PARTS)*.***

***********************************************************************************

***What we know about this case so far:***

***Case description:*** Lucas is a 25-year-old man with cough.

***Quick check for danger signs:***  No danger signs.

***Focused history:***  Cough x 1/12, with night sweats and fatigue. No hemoptysis or weight loss. Previously healthy; never had any serious illness of any kind. No prior treatment for the present illness except for paracetamol. Never tested for HIV, but has multiple current sexual partners.

***Focused physical exam:***  T 38.1 axillary, RR 18, Pulse 90/regular, BP 112/74, BMI 18.4, weight 65 kg. General: No abnormal findings. Neuro: Awake and alert, no deficits. Head, eyes, ears, nose, throat: No abnormal findings. Lungs: inspiratory and expiratory rhonchi over the left posterior lung field. Cardiac and abdominal exams: No abnormal findings.

***Laboratory investigation:*** 2 of 2 sputum smears positive (++) for AFB. HIV antibody testing negative. A malaria slide shows no *P falciparum*.

******************************************************************************

***New information about the case*:**

After the initial visit, Lucas was started on treatment with Isoniazid 75 mg + rifampicin 150 mg + pyrazinamide 400 mg + ethambutol 275 mg, 4 tablets of the fixed-dose combination pill per day every day. He also receives pyridoxine. A community-based treatment supporter was identified to assist him.

LUCAS NOW RETURNS FOR FOLLOW-UP, 2 MONTHS AFTER INITIATION OF TREATMENT.

2 AFB sputum smears are positive (2+) today. His cough and fever have resolved. His weight is increasing. He has no new symptoms except that his urine is now a reddish orange. No other urinary symptoms; urinalysis is normal today.

******************************************************************************

| **Question 7.** Rose, a clinical officer, evaluates Lucas and his laboratory results. What should Rose with with Lucas’s medications now, based on the clinical information reported above? | For scorers only. | |
| --- | --- | --- |
| **Answer to question 7:**   1. **___________________________________________________________** 2. **___________________________________________________________** 3. **___________________________________________________________** |  |  |

| **Question 8. How should Rose manage the reported discoloration of the urine?** | For scorers only. | |
| --- | --- | --- |
| **Answer to question 8:**   1. **___________________________________________________________** |  |  |

| **Question 9.** When should Rose ask Lucas to come back for his next follow-up visit with her? | For scorers only. | |
| --- | --- | --- |
| **Answer to question 9:**   1. **___________________________________________________________** |  |  |

| **Question 10.** If Lucas is still stable at the next visit, what is the single most important thing that Rose should do to evaluate him then? | For scorers only. | |
| --- | --- | --- |
| **Answer to question 10:**   1. **___________________________________________________________** |  |  |

**End of part 2*.***

***Please fold this part of the case and place it in the envelope. Then open the binder and remove the next part of the case, and close the binder. Remember: You may have only one section of the case open at a time. You cannot go back and look at earlier sections of the case once you have placed them in the envelope.***

***IDCAP***

***Scenario 1***

***Part 3***

**Beginning of part 3 (OF 4 PARTS)*.***

***********************************************************************************

***What we know about this case so far:***

**AT THE FIRST VISIT:**

***Case description:*** Lucas is a 25-year-old man with cough.

***Quick check for danger signs:***  No danger signs.

***Focused history:***  Cough x 1/12, with night sweats and fatigue. No hemoptysis or weight loss. Previously healthy; never had any serious illness of any kind. No prior treatment for the present illness except for paracetamol. Never tested for HIV, but has multiple current sexual partners.

***Focused physical exam:***  T 38.1 axillary, RR 18, Pulse 90/regular, BP 112/74, BMI 18.4, weight 65 kg. General: No abnormal findings. Neuro: Awake and alert, no deficits. Head, eyes, ears, nose, throat: No abnormal findings. Lungs: inspiratory and expiratory rhonchi over the left posterior lung field. Cardiac and abdominal exams: No abnormal findings.

***Laboratory investigation:*** 2 of 2 sputum smears positive (++) for AFB. HIV antibody testing negative. A malaria slide shows no *P falciparum*.

***Treatment:*** Lucas was started on treatment with Isoniazid 75 mg + rifampicin 150 mg + pyrazinamide 400 mg + ethambutol 275 mg, 4 tablets of the fixed-dose combination pill per day every day. He also receives pyridoxine. A community-based treatment supporter was identified to assist him.

2 MONTHS AFTER INITIATION OF TREATMENT.

2 AFB sputum smears were positive (2+). His cough and fever had resolved. His weight was increasing. He had no new symptoms except that his urine is now a reddish orange. No other urinary symptoms; urinalysis is normal today.

******************************************************************************

***New information about the case*:**

At the 2-month visit, Rose sent Lucas home on exactly the same medications that he had already taken for 2 months, except for the paracetamol.

**1 MONTH LATER (3 MONTHS AFTER INITIATION OF TREATMENT)**

Lucas returns for follow-up. He reports that he has been vomiting. He seems confused.

This time, 2 of 2 AFB sputum smears are negative. A repeat HIV serology is also negative.

******************************************************************************

| **Question 11.** Which of Lucas’s TB medications might cause Lucas to have vomiting and confusion? Name up to 4. | For scorers only. | |
| --- | --- | --- |
| **Answer to question 11:**   1. **_____________________________________________________** 2. **_____________________________________________________** 3. **_____________________________________________________** 4. **_____________________________________________________** |  |  |

| **Question 12.** If adverse reaction is the cause of Lucas’s confusion and vomiting, what abnormal findings might Rose find on physical examination? Name up to 3 abnormal findings. | For scorers only. | |
| --- | --- | --- |
| **Answer to question 12:**   1. __________________________________________________ 2. __________________________________________________ 3. __________________________________________________ |  |  |

**End of part 3*.***

***Please fold this part of the case and place it in the envelope. Then open the binder and remove the next part of the case, and close the binder. Remember: You may have only one section of the case open at a time. You cannot go back and look at earlier sections of the case once you have placed them in the envelope.***

***IDCAP***

***Scenario 1***

***Part 4***

**Beginning of part 4 (OF 4 PARTS)*.***

***********************************************************************************

***What we know about this case so far:***

**AT THE FIRST VISIT:**

***Case description:*** Lucas is a 25-year-old man with cough.

***Quick check for danger signs:***  No danger signs.

***Focused history:***  Cough x 1/12, with night sweats and fatigue. No hemoptysis or weight loss. Previously healthy; never had any serious illness of any kind. No prior treatment for the present illness except for paracetamol. Never tested for HIV, but has multiple current sexual partners.

***Focused physical exam:***  T 38.1 axillary, RR 18, Pulse 90/regular, BP 112/74, BMI 18.4, weight 65 kg. General: No abnormal findings. Neuro: Awake and alert, no deficits. Head, eyes, ears, nose, throat: No abnormal findings. Lungs: inspiratory and expiratory rhonchi over the left posterior lung field. Cardiac and abdominal exams: No abnormal findings.

***Laboratory investigation:*** 2 of 2 sputum smears positive (++) for AFB. HIV antibody testing negative. A malaria slide shows no *P falciparum*.

***Treatment:*** Lucas was started on treatment with Isoniazid 75 mg + rifampicin 150 mg + pyrazinamide 400 mg + ethambutol 275 mg, 4 tablets of the fixed-dose combination pill per day every day. He also receives pyridoxine. A community-based treatment supporter was identified to assist him.

**2 MONTHS AFTER INITIATION OF TREATMENT.**

2 AFB sputum smears were positive (2+). His cough and fever had resolved. His weight was increasing. He had no new symptoms except that his urine was a reddish orange, No other urinary symptoms; urinalysis was normal. Rose (a clinical officer) sent Lucas home on exactly the same medications that he had already taken for 2 months, except for the paracetamol.

**1 MONTH LATER (3 MONTHS AFTER INITIATION OF TREATMENT)**

Lucas returned for follow-up. He reported that he has been vomiting.

******************************************************************************

***New information about the case (continuation of 3-month follow-up visit described in part 3):***

***New history:***  Lucas has vomited 3 or more times per day for last 5 days. Abdomen is painful. Very tired. No fever, no cough, no other symptoms. Has taken his medications faithfully (treatment card confirms).

***New physical exam:***  Vital signs normal, but appears confused and slightly dehydrated. Weight 63 kg. Yellow sclera. Abdomen: Right upper quadrant tenderness with liver enlargement with liver tenderness. No other danger signs encountered.

******************************************************************************

| **Question 13.** What is the single most probable cause of Lucas’s signs and symptoms? | For scorers only. | |
| --- | --- | --- |
| **Answer to question 13:**   1. **___________________________________________________________** |  |  |

| **Question 14. How should Rose manage Lucas’s TB medications today?** | For scorers only. | |
| --- | --- | --- |
| **Answer to question 14:**   1. **__________________________________________________________** 2. **__________________________________________________________** |  |  |

| **Question 15. If available, what laboratory test or tests should Rose order to confirm the diagnosis that she suspects? Name up to 3 tests.** | For scorers only. | |
| --- | --- | --- |
| **Answer to question 15:**   1. **_________________________________________________** 2. **________________________________________________** 3. **________________________________________________** |  |  |

| **Question 16. Is TB treatment failure the most likely cause of Lucas’s signs and symptoms? Y/N** | For scorers only. | |
| --- | --- | --- |
| **Answer to question 17: _____** |  |  |

**END OF CASE SCENARIO.**

***Place this scenario in the envelope with your name on it and close the envelope before going on to the next scenario. If this is your last scenario of the day, give your binder and envelope to the facilitator before leaving.***
