# Supplementary material for: Capacity-Building and Clinical Competence in Infectious Disease in Uganda: A Mixed-Design Study with Pre/Post and Cluster-Randomized Trial Components
Source: PLoS One. 2012 Dec 14;7(12):e51319. doi: 10.1371/journal.pone.0051319 (PMC3522731; doi:10.1371/journal.pone.0051319)
Supplement: Protocol S1 — Trial Protocol. (DOC) [file pone.0051319.s002.doc]

**Project Title**: Integrated Infectious Disease Capacity-Building Evaluation (IDCAP)

**Study Title**: Cost effectiveness of building capacity of mid-level practitioners in Sub-Saharan Africa for the care and prevention of HIV, Tuberculosis, Malaria and related infectious diseases

**Principal Investigator**

Marcia Weaver, PhD

Research Associate Professor, Department of Global Health

International Training and Education Center on HIV (I-TECH)

University of Washington

901 Boren St, Suite 1100

Seattle, WA 98104

[mweaver@uw.edu](mailto:mweaver@uw.edu)

USA office (206) 616-9173;

Uganda mobile (256) 753-713-914

**Site Principal Investigators and co-Principal Investigators**

Sarah Naikoba

BMBCh, MPH, MSc (Candidate)

Head of Training Department, Infectious Diseases Institute

Makerere University

Plot 15 (or 17) Kitante Road

Kampala Uganda

[snaikoba@idcap.org](mailto:snaikoba@idcap.org), [naikobasarae@gmail.com](mailto:naikobasarae@gmail.com)

Office (256) 312-211-422

Mobile (256) 712 589 002

Lydia Mpanga Sebuyira

BM BCh, BA (Hons) Physiological Sciences, MA (Oxon), FRCP

Senior Scientist, Infectious Diseases Institute

Faculty of Medicine

Makerere University

P.O. Box 22418

Kampala Uganda

lmsebuyira@imprintld.com

Mobile (256)-755-702266

**November 2011**

# ABBREVIATIONS

| ART | Antiretroviral Therapy |
| --- | --- |
| ATIC | AIDS Treatment Information Center |
| CHOICE | Choosing Interventions that are Cost-Effective |
| CQI | Continuous Quality Improvement |
| DALY | Disability Adjusted Life Year |
| DHS | Demographic and Health Survey |
| EA | Enumeration Area |
| HCI | Health Care Improvement Project |
| IDI | Infectious Diseases Institute |
| IDT | Infectious Diseases Training |
| IMAI | Integrated Management of Adult and Adolescent Illness |
| IMCI | Integrated Management of Childhood Illness |
| I-TECH | International Training and Education Center on Health |
| MCT | Multi-disciplinary Clinical Training |
| MLP | Mid-level practitioner |
| OSS | On-site support services |
| PEPfAR | President’s Emergency Plan for AIDS Relief |
| SOMREC | School of Medicine Research and Ethics Committee |
| U5 | Child less than 5 years |
| UBOS | Uganda Bureau of Statistics |
| USAID | United States Agency for International Development |
| WHO | World Health Organization |

# DEFINITION OF OPERATIONAL TERMS

Efficacy - Efficacy describes how well treatment works in ideal circumstances

Effectiveness - Effectiveness describes how well treatment works in real life. Treatments may work less well in practice than clinical settings due to patient non-compliance and co-morbidity

Cost-effective - Compares incremental cost to incremental effectiveness, e.g. $/life years saved from intervention vs. usual care

Incremental Cost-Effectiveness Ration (ICER) – ICER is the ratio of the difference in cost between two group of subjects and the difference in effectiveness between two groups of subjects.

Task shifting - According to WHO, task shifting is the rational redistribution of tasks among health workforce teams, “Specific tasks are moved, where appropriate, from highly qualified health workers to health workers with shorter training and fewer qualifications in order to make more efficient use of available human resources for health.”

Mid-level practitioners – These practitioners are defined in this proposal as clinical officers, nurses, and midwives.

Clinical officers – These non-physician clinicians have a secondary school education (13 years), three years of pre-service training and two years of internship.

Registered Nurses or Midwives – These nurses and midwives have a secondary school education (13 years) and three years of pre-service training

Enrolled Nurses or Midwives – These nurses and midwives have a secondary school education (13 years), and one and one-half years of pre-service training.

Nurse-midwives - These double-trained professionals have a secondary school education (13 years) and four and one-half years of pre-service training.

TABLE OF CONTENTS

[**ABBREVIATIONS 2**](#__RefHeading___Toc244140755)

[DEFINITION OF OPERATIONAL TERMS 3](#__RefHeading___Toc244140756)

[TABLES, FIGURES & BOXES 5](#__RefHeading___Toc244140757)

[ABSTRACT 6](#__RefHeading___Toc244140758)

[PREFACE 7](#__RefHeading___Toc244140759)

[CHAPTER ONE: INTRODUCTION 8](#__RefHeading___Toc244140760)

[1.1 Introduction 8](#__RefHeading___Toc244140761)

[1.2 Literature review 8](#__RefHeading___Toc244140762)

[CHAPTER TWO: RESEARCH PROBLEM 10](#__RefHeading___Toc244140763)

[2.1 Problem Statement 10](#__RefHeading___Toc244140764)

[2.2 Significance 10](#__RefHeading___Toc244140765)

[2.3 Research Question 11](#__RefHeading___Toc244140766)

[2.4 Objectives 11](#__RefHeading___Toc244140767)

[2.4.1 General ‘ 11](#__RefHeading___Toc244140768)

[2.4.2 Specific objectives 11](#__RefHeading___Toc244140769)

[2.5 Hypotheses 12](#__RefHeading___Toc244140770)

[CHAPTER THREE: METHODOLOGY 13](#__RefHeading___Toc244140771)

[3.0 Overview 13](#__RefHeading___Toc244140772)

[3.1 Site Assessment 15](#__RefHeading___Toc244140773)

[3.1.0 Objectives 15](#__RefHeading___Toc244140774)

[3.1.1 Methods 15](#__RefHeading___Toc244140775)

[3.1.2 Design 16](#__RefHeading___Toc244140776)

[3.1.3 Setting 16](#__RefHeading___Toc244140777)

[3.1.4 Population 16](#__RefHeading___Toc244140778)

[3.1.5 Ethical considerations 17](#__RefHeading___Toc244140779)

[3.1.6 Limitations 17](#__RefHeading___Toc244140780)

[3.2 Competence and practice of MLP 18](#__RefHeading___Toc244140781)

[3.2.1 Methods 18](#__RefHeading___Toc244140782)

[3.2.2 Design 18](#__RefHeading___Toc244140783)

[3.2.3 Setting 18](#__RefHeading___Toc244140784)

[3.2.4 Population 19](#__RefHeading___Toc244140785)

[3.2.5 Ethical Considerations 19](#__RefHeading___Toc244140786)

[3.2.6 Limitations 20](#__RefHeading___Toc244140787)

[3.3 Facility Cost and Performance Indicators 22](#__RefHeading___Toc244140788)

[3.3.1 Methods 22](#__RefHeading___Toc244140789)

[3.3.2 Design 26](#__RefHeading___Toc244140791)

[3.3.3 Setting 27](#__RefHeading___Toc244140793)

[3.3.4 Population 27](#__RefHeading___Toc244140794)

[3.3.5 Ethical Considerations 28](#__RefHeading___Toc244140795)

[3.3.6 Limitations 30](#__RefHeading___Toc244140796)

[3.4 Mortality Survey 32](#__RefHeading___Toc244140797)

[3.4.1 Methods 32](#__RefHeading___Toc244140798)

[3.4.2 Design 32](#__RefHeading___Toc244140799)

[3.4.3 Setting 33](#__RefHeading___Toc244140800)

[3.4.4 Population 33](#__RefHeading___Toc244140801)

[3.4.5 Ethical considerations 34](#__RefHeading___Toc244140802)

[3.4.6 Limitations 35](#__RefHeading___Toc244140803)

[REFERENCES 36](#__RefHeading___Toc244140804)

# TABLES, FIGURES & BOXES

Table 1: Summary of Indicators 14

Table 2: Example of calculations of the incremental cost-effectiveness ration of a capacity building program for co-trimoxazole prophalaxis 24

Table 3: Examples of published estimates of cost effectiveness and facility performance indicators 25

Table 4: Sample size calculations to test the effect of the training program on malaria indicators 27

Table 5: Sample size calculations for mortality data 31

Figure 1: Evaluation design 13

Box 1: Uganda’s public health facilities 10

# ABSTRACT

This evaluation aim is to investigate a cost-effective way to build capacity for the care and prevention of infectious diseases among mid-level practitioners in sub-Saharan Africa. Classroom based training continues to be the dominant form of training, despite evidence that suggests that on-site support (OSS) is more beneficial. Definitive evidence that on-site support is the most effective way to deliver the required outputs and related outcomes is still lacking. IDCAP will provide two interventions that integrate training in TB, HIV/AIDS and malaria as well as other infectious diseases, and the effects will be studied.

**Infectious Disease Training (IDT)** - An integrated core curriculum for MLP in the comprehensive care and prevention of infectious disease, delivered to 72 MLP from 36 health facilities at IDI in three sessions for a total of five weeks with extensive clinical sessions.

**On-Site Support Services (OSS)** – CQI activities and a set of responsive Multidisciplinary Clinical Training (MCT) modules for use at the clinic level with teams of medical officers, clinical officers, nurses and midwives, laboratory technicians and records keepers will be sequentially delivered to the 36 facilities in two phases. Sites will be randomly assigned to either Phase 1 or 2.

The evaluation of the capacity-building program will assess the impact of IDT on individual performance and impact of OSS on cost, clinic performance and health outcomes, and definitively test whether or not the incremental impact of on-site support services relative to classroom training alone can be cost-effective. The four alternative hypotheses are:

1. An Integrated IDT course for mid-level practitioners is effective at building the skills required to perform key clinical tasks at acceptable standards
2. Onsite Multidisciplinary Team training and Continuous Quality Improvement activities (or On-Site Support- OSS) significantly improve clinic performance
3. OSS activities are cost effective
4. After monthly OSS for nine months during Phase 1 at Phase 1 sites, bi-monthly OSS for six months has an additional effect on facility performance

For hypothesis 1, the unit of analysis will be the individual trainee. The effect of IDT on capacity and practice will be measured with a pre/post comparison and by comparing the performance of the trainees at the end of the training program to an objective standard.

For hypotheses 2-4, the unit of analysis will be the site. The effect of OSS on cost, clinical performance and health outcomes will be measured by a comparison of Phase 1 and Phase 2 sites.

**Significance**: IDCAP will provide a model of an integrated capacity-building package for the care and prevention of infectious disease in Uganda and Africa at large. In Uganda, the 72 trained MLP and 432 health professionals at 36 sites will serve about 17% of Uganda’s population. The evaluation will contribute to current literature on the role of MPL in the management of infectious diseases and cost effective approaches to capacity building of MLP. Results will inform donors and governments on investments in human resource development in the health sector.

# PREFACE

The IDCAP proposal was submitted to the Bill and Melinda Gates Foundation three times beginning in spring 2007. The final version was submitted in March 2008 and approved for funding in November 2008. The proposal followed the Foundation’s format and was organized around five main activities, rather than the format of scientific proposals submitted to organizations such as the United States National Institutes of Health.

IDCAP is an evaluation of a training and quality improvement program. At the time the proposal was submitted, it was not envisioned that the evaluation would require human subjects/ethics committee review. The University of Washington’s Humans Subjects Review Committee made a similar determination.

In January 2009, the Uganda National Committee on Science and Technology (UNCST) recommended that we submit IDCAP so that they could review the distinction between quality improvement and research in more detail. As part of that process, the School of Medicine at Makerere University Research Ethics Committee (SOMREC) is also respectfully requested to review IDCAP. This proposal was prepared to comply with a SOMREC outline for research proposals.

# CHAPTER ONE: INTRODUCTION

## 1.1 Introduction

Considerable progress continues towards increasing access to anti-retroviral therapy (ART) in resource-limited settings. WHO, UNAIDS, and UNICEF recently estimated that 1.3 million people have access to ART in Sub-Saharan Africa, or 28% of people with HIV living there who need ART.[[1]](#endnote-2) These accomplishments required training of health professionals, among other efforts to strengthen health systems. For example, the United States’ Presidents Emergency Plan for AIDS Relief supported the training of 154,000 health professionals in ART from 2004 to 2007.[[2]](#endnote-3) The greatest challenge to increasing access to ART however, is the shortage of trained health care professionals.Error: Reference source not found [[3]](#endnote-4) [[4]](#endnote-5) [[5]](#endnote-6)

##

## 1.2 Literature review

**Mid-Level Practitioners (MLP) are emerging as the ideal clinicians in Africa.** In 2006, the Infectious Diseases Institute (IDI), in collaboration with the Ugandan Ministry of Health (MOH) conducted a national HIV/AIDS Training Needs Assessment which revealed that responsibilities usually reserved for a doctor, such as prescribing, are often passed on to clinical officers and nurses who report feeling insufficiently trained to carry out such activities. Ninety percent of doctors, 60% of clinical officers, 14% of nurses and 24% of midwives who worked in accredited ART clinics reported that they prescribe ART (p<0.001). Sixty-four percent of the people who prescribed ART were not doctors. Among professionals who prescribed ART, 76% of doctors, 62% of clinical officers, 62% of nurses and 51% of midwives were trained in initiating patients on ART (p=0.457); 73%, 46%, 50%, and 23%, respectively, were trained in monitoring patients on ART (p=0.017). Seven percent of doctors, 42% of clinical officers, 35% of nurses, and 77% of midwives who prescribed ART assessed that their overall knowledge of ART was lower than good (p=0.001).[[6]](#endnote-7)

While such MLP represent a primary segment of the healthcare workforce in Uganda and elsewhere, the medical officer is still most often identified as the recipient of coordinated training activities. With appropriate training, however, MLP have been shown to successfully deliver antiretroviral therapy to HIV patients in Kenya,[[7]](#endnote-8) Malawi,[[8]](#endnote-9) Mozambique,[[9]](#endnote-10) [[10]](#endnote-11) Rwanda,[[11]](#endnote-12) and Zambia.[[12]](#endnote-13) [[13]](#endnote-14) Because MLP are less likely than physicians to migrate toward higher paying jobs in the developed world, have shown competence in delivering services normally reserved for medical officers, and are already providing antiretroviral care in many parts of Africa, they may well be the most valuable existing resource in Africa to deliver ongoing care of infectious diseases.

**Existing training programs are inefficiently aligned with vertical disease programs.** In Uganda, training activities for all cadres of healthcare are often provided by international entities and non- governmental organizations that function independently, outside of the MOH planning framework. Such training efforts are most often conducted through isolated disease-specific programs, creating expensive redundancies even while training programs remain fragmented and incomplete. Even programs like PEPFAR, the Global Fund to Fight AIDS, Tuberculosis and Malaria, and the President’s Malaria Initiative provide support vertically by disease, creating inherent inefficiencies for human capacity-building efforts. And while HIV/AIDS, malaria and tuberculosis combined account for 29% of mortality in developing countries, illnesses such as acute respiratory illness, diarrhoeal disease and measles account for a slightly greater number of deaths (30%).[[14]](#endnote-15)

**Information regarding the most cost-effective way to train MLP to deliver quality care of infectious disease is critically needed.** Despite evidence suggesting that knowledge gained through intensive classroom training courses is not fully applied once the trainee returns to his/her real-world clinical setting,[[15]](#endnote-16) and that on-site training[[16]](#endnote-17) and supervision[[17]](#endnote-18)can favourably impact clinical practice, investment in human capacity-building continues to be concentrated on classroom training with little or no follow-up reinforcement. The status quo persists in part because of the lack of documented cost-effectiveness analysis of more costly on-site support services. Little is done to integrate or evaluate these supplemental training efforts, and training programs vary considerably in course content, duration, target recipient, and delivery mechanisms.

Experts in global health are calling for increased funding for training of MLP, and stressing the need for large donors to incorporate task shifting into their funding plans. Amid recognition that poor adherence to medication and undetected treatment failure can lead to pathogen resistance, however, much care is needed to ensure that MLP are well prepared to provide quality care. The development community seems poised to expand training efforts exponentially to address mid-level healthcare providers – who outnumber doctors in Africa by at least 6 to 18 – without the prerequisite understanding of which training approaches yield the best and most lasting results. Evidence for effective approaches is therefore much needed.

# CHAPTER TWO: RESEARCH PROBLEM

## 2.1 Problem Statement

Little has been done to test the effectiveness of disparate approaches to improving healthcare service delivery through human resource development. As the global health community seeks to expand training efforts to address mid-level healthcare providers (nurses and clinical officers) rigorous evaluation of which training approaches yield the best and most lasting results is needed to know what type of training program to implement or fund.

## 2.2 Significance

**The principal output of this evaluation will be a proven model of an integrated capacity-building package for the care and prevention of infectious disease in Africa.** The package will include a single curriculum for MLP and a portfolio of on-site support services founded on Accordia Global Health Foundation’s considerable experience in training clinicians in North America and Africa.

**IDCAP will train 72 MLP and 432 multidisciplinary team members at 36 sites throughout**

**Uganda.** These sites serve a catchment area of approximately 17% of the country’s population.

**Box 1. Uganda’s Public Health Facilities**

11 Regional Referral Hospitals – 2,000,000 served per hospital

55-70 District Reference Hospitals – 500,000 served per hospital

150-200 Health Center IV (primarily clinical officers and nurses) – 100,000 served per center

900-1000 Health Center III (primarily clinical officers and nurses; no lab services) – 20,000 served per center

**The population in the areas surrounding the proposed sites will benefit directly from lower infection, illness and mortality rates.** The improved ability of clinic staff to deliver quality prevention and care services will increase access to cost-effective interventions delivered according to national guidelines by 9% to 25% depending on the intervention.

**IDCAP will provide definitive evidence on the cost-effectiveness of on-site support.** The results on the cost-effectiveness of on-site support services including Continuous Quality Improvement (CQI) activities and on-site training for multidisciplinary teams) could potentially encourage investment away from up-front classroom-based training programs, and toward the workplace capacity building programs widely suspected to have more long-lasting results.

**Results of this evaluation will enable more efficient use of donor investment.** This evaluation seeks to identify the nature of the ideal capacity-building program for MLP in Uganda, enabling the considerable amount of global resources to focus on its replication throughout Africa and the developing world. Large donors will be encouraged to support the whole or a portion of an integrated infectious disease training program rather than disease-specific training approaches currently being employed. Demonstration of achieved competence in treating and preventing multiple infectious diseases will hopefully dissuade donors from continuing the currently inefficient and redundant efforts found in disease-specific training programs.

## 2.3 Research Question

Two sets of questions will be addressed.

1) Is an Integrated ID Training course (classroom and distance learning) for mid-level practitioners effective at building the skills required to perform key clinical tasks at acceptable standards? To answer this question, 2 mid-level practitioners from each of 36 sites will be invited to a Core Infectious Diseases course at IDI. The unit of analysis will be the individual. The evaluation design will be a before/after comparison of trainee skills and performance.

2) Does Onsite Multidisciplinary Team training and Continuous Quality Improvement activities, which is called On-Site Support (OSS), significantly improve clinic performance and health outcomes? If yes, are the OSS activities cost-effective? To answer these questions, 36 sites will receive the OSS activities for nine months, but 18 will be randomly assigned to receive the OSS in phase 1 and the others will receive it nine months later. The unit of analysis will be the site, and the evaluation design is a randomized trial of sites on key performance indicators.

## 2.4 Objectives

### 2.4.1 General ‘

To evaluate the effectiveness of an integrated training program for mid-level practitioners on the care and prevention of infectious diseases, and the incremental impact and cost-effectiveness of on-site support services, through their randomized and staggered introduction at 36 sites in Uganda in order to identify a cost-effective way to build capacity of mid level practitioners in sub-Saharan Africa for the care and prevention of infectious diseases

### Specific objectives

1. Evaluate the effectiveness of the integrated Infectious Disease Training in preparing mid-level practitioners to perform key clinical tasks corresponding to infectious disease competencies at acceptable standard
2. Evaluate the effectiveness of on-site support services (OSS) in improving clinic performance
3. Estimate the cost-effectiveness of site support services
4. Compare two methods for estimating cost-effectiveness: a) economic modeling in which effectiveness is measured by original data on performance indicators at 36 sites and published estimates of the effect of the performance indicators on Disability Adjusted Life Years (DALYS), and b) estimates in which effectiveness is measured original verbal autopsy data on mortality among children five years or less.
5. Evaluate the effectiveness of bi-monthly OSS for six months on clinical performance at Phase 1 sites after monthly OSS for nine months during Phase 1.

##

## Hypotheses

1a. **Null:** Among MLP, there will be no difference before and after attending the IDT in skills competence or practice at performing clinical tasks for infectious diseases.

1b. **Alternative**: MPL who attend the IDT will significantly increase their competence and practice at performing clinical tasks for infectious diseases. Further the increase will demonstrate competence and practice to meet objective standards.

2a **Null**: There will be no difference in facility performance between the multi-disciplinary teams at 18 sites that receive OSS in Phase 1 and those at 18 sites that receive OSS in Phase 2.

2b **Alternative**: On-site support for multi-disciplinary teams will significantly improve facility performance among the 18 Phase 1 sites relative to the 18 Phase 2 sites.

3a **Null**: Even if OSS activities are effective, they are too expensive to justify scale-up.

3b **Alternative**: OSS activities are cost-effective relative to interventions that are currently recommended by international organizations, such as the WHO.

4a **Null:** The results of the cost-effectiveness analysis are the same regardless of whether the effectiveness is measured by economic modeling or original data on mortality.

4b **Alternative:** The cost-effectiveness ratio based on original data on mortality will be lower and consequently more favorable than the ratio based on economic modeling.

5a **Null**: After monthly OSS for nine months during Phase 1 at Phase 1 sites, bi-monthly OSS for six months during Phase 2 has no effect.

5b **Alternative:**  performance at Phase 1 sites during Phase 1, ii) contemporaneous performance at Phase 2 sites, and 3) performance at Phase 2 sites in the absence of OSS in Phase 2 as extrapolated from their performance in Phase 1.

# CHAPTER THREE: METHODOLOGY

## 3.0 Overview

##

There are four major components of the evaluation design as described below: 1) Site Assessment, 2) Competence and practice of individual MLP who participate in IDT, 3) Facility cost and performance indicators, and 4) Mortality survey. The site assessment guided the curriculum development and site selection. As shown in Figure 1, it occurred only once in Year 1. The competence and practice of individual MLP will be evaluated with methods that are currently used by the Infectious Diseases Institute (IDI) to evaluate trainees. Data on MLP will be collected three times: a) before training, b) after the three-week course, and c) after the nine-month program that includes two, one-week booster courses and distance learning. Facility performance indicators will be collected on a monthly basis from each site. Data for external quality assurance of laboratory performance will be collected at baseline and end of the intervention or analyzed as secondary monthly data from the Tuberculosis National Reference Laboratory. Sites will be randomly assigned to begin OSS in either Phase 1 or Phase 2 and the facility indicators of these two samples of sites will be compared. The principal investigators of IDCAP are actively considering collecting data on mortality among children under five, but a final decision has not been made. At the request of the acting chairperson of SOMREC, we are including information about this potential activity. Mortality surveys would be conducted before and after the OSS. In this chapter, each component of the evaluation is described separately.

**Figure 2: Evaluation Design**

The indicators that will be collected from components 2-4 are summarized in Table 1. No indicators were collected for the Site Assessment.

|  | **Table 1 Summary of Indicators** | | | |  | | | | | |
| --- | --- | --- | --- | --- | --- | --- | --- | --- | --- | --- |
|  | | Hypothesis 1: Build clinical skills of core course trainees | | Hypotheses 2-4: Test effectiveness of on-site multidisciplinary training and continuous quality improvement | | | | | | |
|  | | Com-petency | Practice | Process indicators | | Laboratory Performance | Clinical Performance (examples) | Modeling health outcomes | | Direct measure of health outcomes or mortality |
|  |  |  |  |  |  |  |  | Effective Coverage | Cost-effective intervention (from literature) |  |
| HIV prevention | | Measured by case studies or case scenarios | Measured by observation of trainees at their site | 1) Staff on-site  2) Employee engagement,  3) Availability of supplies, including   - HIV test kits - cotrimoxazole - first line ART - TB drugs - coartem - reagents for laboratory tests | | HIV test  DBS | % HIV positive pregnant women with an HIV/ART card  % HIV exposed infants on CTX | Number of pregnant women with HIV who are referred to PMTCT | PMTCT | HIV exposed children who are HIV- |
| HIV care | |  |  |  |  | Blood test  Urine test  CD4 specimen | Number of HIV+ patients prescribed daily cotrimoxazole | Number of HIV+ patients on cotrimoxazole therapy | Daily Cotrimoxazole | Mortality of HIV patients |
| ART | |  |  |  |  |  | Number of ART naïve HIV+ patients who are eligible for ART who start 1st line ART | Number of HIV patients on ART | ART | Mortality of ART patients |
| TB/HIV | |  |  |  |  | HIV test  Sputum smear | Number of TB suspects with known HIV status  Number of TB/HIV patients started on ART within 3 months of starting TB treatment | Number of patients who are smear negative or extra-pulmonary TB detected & cured under DOTS | Full DOTS | Mortality of TB/HIV patients |
| Case mgmt of respiratory illness | |  |  |  |  | Sputum smear | Number of TB suspects sent for first AFB smear  Number of smear negative TB suspects started on empiric treatment | Number of TB patients detected and cured under DOTS  Number of children diagnosed with pneumonia | - DOTS - Case mgmt of pneumonia for children | Mortality of TB patients  and children under 5 |
| Case mgmt of fever | |  |  |  |  | Malaria smear | % of malaria suspects sent for blood smear  % of smear negative patients treated with any antimalarials | Number of patients with a positive laboratory test for malaria (blood smear) who are treated with ACT | Case management with ACT | Mortality of children under 5 |
| Emer-gency treatment and triage | |  |  |  |  |  | Number of patients triaged on a random sample of days |  |  | Mortality within 24 hrs of admission |

# 3.1 Site Assessment

### 3.1.0 Objectives

1. Establish the background knowledge of mid-level health practitioners in malaria, tuberculosis and HIV/AIDS
2. Establish the range of tasks performed by mid-level health practitioners with regard to management of malaria, tuberculosis and HIV/AIDS
3. Assess the quality of recording of data in the various registers used by the health centre
4. Document available facilities that support On-Site Training
5. Identify health facilities that have two clinical officers
6. Identify health facilities that have a functional laboratory that can perform basic tests

### 3.1.1 Methods

Primarily quantitative techniques of data collection were employed in this assessment. This was complimented with key informant interviews and document review. Data was collected from all cadres of health professionals present at the facility including some who were off-duty for the day. A total of five different tools were administered at every visited site. Facility Checklist (observation):

- HMIS data quality guide
- Learning needs assessment guide
- Laboratory personnel assessment guide
- Facility laboratory assessment guide
- Facility in-charge key informant guide

The key documents reviewed include the staff list, HMIS105 & 108 report forms, comprehensive HIV including ART monthly reports from every facility visited as well as all registers in use at the facilities.

Prior to the assessment, the questionnaires were pre-tested in three Kampala City Council (KCC) clinics in Kampala. The pre-test was among others used to assess consistency, accuracy and feasibility of the questionnaires and before the main assessment was conducted. The pre-test also helped gather information on comprehension of the questions.

Five teams of graduate researchers consisting of one Laboratory Technician and two Social Scientists administered the tools at the selected sites. The teams underwent a rigorous one-day training sessions focusing on the objectives of the assessment, assessment design and data collection.

Structured questionnaires and key informant interview guides served as the primary tools for data collection. Information regarding data/records was obtained from the registers with the help of the persons that manage them most of the time. Data on the completeness of the records rather than the patient information were recorded. Data on clinic performance and standards of care were obtained from HMIS 105&108 report forms.

### 3.1.2 Design

This assessment adopted a descriptive design employing quantitative methods of data collection as well as key informant interviews with the in-charge at the site. Structured questionnaires and key informant interview guides served as the primary tools for data collection. Review of facility reports and registers was also done.

### 3.1.3 Setting

A total of 45 purposively selected health facilities (sites) were visited across the country. The sites were drawn from all the six major regions of the country i.e. West Nile, Northern Uganda, Eastern Uganda, Central, Western, and South Western Uganda. The sites visited were a mix of Hospitals, Health Center (HC) IVs and HC IIIs.

The sites for assessment were purposively selected following a well laid out criteria. A listing of all health facilities meeting the criteria was obtained from MoH’s Quality of Care Initiative. The list was developed following the outlined criteria below:

- The health facility had to be a HC IV or comparable facility such as a small general hospital, large HC III or non-governmental organization clinic
- It had to be an active ART site or accredited ART site that is scheduled to become active by April 2009
- No past or current participation in MoH quality improvement programs such as Health Care Improvement (HCI) Project or HIVQual
- No past or current participation in a partnership with the U.S. Department of Defense
- Has a population of potential patients who are not prisoners
- Representation from all six administrative regions of Uganda

Reconnaissance calls to confirm the operational status of the health facility were made to the potential sites prior to the assessment. All the 45 sites visited met the inclusion criteria outlined above.

### 3.1.4 Population

At every site visited attempts were made to adopt total coverage of all the health professionals available on site. Participants in the assessment included doctors, clinical officers, nurses, midwives, nursing assistants and laboratory personnel, among others. An inventory of staff for the various sites was obtained from the District Health Office (DHO), verified on-site by the head of the facility, and was used to calculate the response rate for the learning needs assessment for clinical and laboratory staff. Those who did not participate were either on study leave, off-duty or out of station.

At the selected sites, the first contact was the in-charge of the health centre / hospital or his/her designees. This person served as the key informant who provided general information about the site including the number of available clinical officers. The head of the laboratory was in turn asked to help with the assessment for the functionality of the laboratory.

### 3.1.5 Ethical considerations

**Background**

The site assessment was completed before IDCAP was reviewed by the Makerere University Faculty of Medicine Research Ethics Committee (SOMREC) and Uganda National Committee on Science and Technology (UNCST). The assessment collected descriptive data to guide curriculum development and site selection. The principal investigators did not consider it research, because no hypotheses were tested. At the request of SOMREC, this component of the evaluation is included in the proposal.

**Consent**

The evaluators sought informed consent from the participants as a best practice for any endeavor that involves collecting data from human beings. Verbal informed consent was requested from each participant. In the case of the Learning Needs Assessment and Key Informant Interview, there was a formal process in which participants were informed that the interviews were voluntary and confidential. “Confidential” means the participants names were not be linked the data that are entered into the database and only aggregate results were presented.

**Data storage**

No human names were recorded on any of the questionnaires and facility name was only recorded on the Key Informant Interview Guide. All sites were identified by a site number on all questionnaires except the Key Informant Guide; it would not be possible to identify individual participants from those paper forms. The key linking the site number to the facility name was kept securely.

Participants were informed that the data were confidential rather than anonymous, because a determined person who had access to the key might be able to identify an individual respondent if s/he was the only person who reported a specific profession at a health facility.

### 3.1.6 Limitations

Training needs as measured by previous training and self-assessment of competence. A review of studies of health professionals in the United States and Europe concluded that the validity of self-assessment of performance was low to moderate. Consequently, the assessment provides only a gross estimate of training needs. More detailed information based on observation of practice by the clinical mentors and more innovative method such as trainee diaries, in which health professionals note difficult situations during patient encounters.[[18]](#endnote-19)

Also, the health professionals were a convenience sample of those who were at the sample of accredited ART clinics on the day of the assessment. Consequently the sample over represented some types of professionals at some facilities and under-represented others; doctors were underrepresented at all facilities.

# 3.2 Competence and practice of MLP

### 3.2.1 Methods

The first specific objective is to “evaluate the effectiveness of the integrated Infectious Disease Training in preparing mid-level practitioners to perform key clinical tasks corresponding to infectious disease competencies at acceptable standard.” Two mid-level practitioners from each of 36 sites will be invited to a Core Infectious Diseases course at IDI. The unit of analysis will be the individual. The evaluation design will be a before/after comparison of trainee skills and performance.

Individual trainees will complete eight to 12 written case scenarios and be observed in clinical practice at three times during the core course: Pretest, post 3-week session, post 9-month classroom and distance program.

IDI routinely evaluates the knowledge of its trainees with multiple choice tests and clinical practice with observation of trainees at the Adult Infectious Diseases Clinic. Trainees must obtain a passing score on these activities to be awarded a certificate. Trainee scores are confidential. Trainees can retake the knowledge test and observation of clinical practice until they pass. These evaluations are similar to any educational program that grants diplomas or degrees on the basis of assessment of student/trainee accomplishments and are generally exempt from ethical review.

The IDCAP evaluation is the same as the routine IDI evaluation in the sense that trainees must obtain a passing score to be awarded a certificate and trainee scores will be confidential. It differs from the routine IDI evaluation in the methods of assessment: 1) the knowledge test will require answers to open-ended questions rather than multiple choice questions, and 2) observation of clinical skills will occur at the trainee’s health facility rather than at the Adult Infectious Diseases Clinic.

### 3.2.2 Design

The alternative hypothesis that IDT has a significant effect on trainee competence and practice will be tested with a before/after comparison. The indicators of infectious disease competencies at baseline will be compared to those measures at the end of each session of the IDT core and booster courses. The indicators will be used to establish an acceptable standard, and we will test for differences in the percentage of MLP who meet the standard between baseline and subsequent measures. In regression analysis, we will adjust for unobserved differences across MLP using a random effects model and the *xtgee* command in Stata version 10. We will also compare change in competence between Arms A and B to test the incremental impact of OSS

on the proportion of MLP who meet the standards.

### 3.2.3 Setting

Case scenarios will be completed at IDI. The observation of clinical practice will occur at the facility where the trainee works. See Section 3.1.3 for a list of the site selection criteria.

### 3.2.4 Population

Participants will be MLP who enroll in the Core IDT. Calculations of the number of participants required were based on the average scores of the pretest (42%) and post test (62%) for the Comprehensive HIV Care Including ART course during 12 separate occasions when it was taught at IDI. The standard deviation of scores for all participants was .11. The standard deviation of scores within groups at each occasion when the course was taught was .12 and the correlation between before and after test scores within a group was 0.5.

There are two approaches to considering sample size here, both of which are simplified versions of the analysis. The first is a single sample of individuals, who are tested before and after the three-week Core IDT. Participants from phase 1 and phase 2 sites are not expected to differ, because OSS has not started at the Phase 1 sites. A sample size of 13 would be needed to detect a change in average test score from .4 (pretest) to .5 (post test).

The second is a comparison of two groups: participants from phase 1 site and participants from phase 2 sites. At the end of the nine-month after IDT, participants from the phase 1 sites will have also benefited from OSS and their scores would increase more than participants from phase 2 sites. Each group has an average before test score (either pretest or after the three-week Core IDT) and an average nine-month test score and hence an average of differences in scores between before and nine-months. The effect will be the difference in average differences between the groups. A sample size of 23 participants in each group would be needed to detect a difference in change in scores of .1.

### Based on IDI’s experience with the Joint Uganda Malaria Training Program (JUMP), the Core IDT was designed for two participants from each site. As explained in Section 3.3.4 with the sample size calculations for the facility cost and performance indicators, a sample size of 18 sites per treatment group or total of 36 sites was required. Consequently 72 participants would attend the Core IDT course. The evaluation is adequately powered to assess changes in case scenario scores, particularly as we have observed changes of double the magnitude that were used in the sample size calculations.

###

### 3.2.5 Ethical Considerations

**Consent**

Each trainee will apply to the IDI. The application form has two parts: a) course application, and b) consent to share the trainees’ data with the IDCAP evaluation. For the consent form, permission is requested to use trainee data to create a confidential record that will be used for the IDCAP evaluation. As stated on the form, “The decision is voluntary. You should feel completely free to choose not to share the data and to withdraw your data from the analysis without giving a reason.”

During the two days of clinical observation, a representative at each site and an IDCAP clinician will introduce the activity to the patients in the waiting room. Patients will be asked to give verbal consent to participate. The patient data will be anonymous, and the verbal consent process will preserve their anonymity. If the clinic staff need to distinguish patients who consent from other, the former will receive a card at the registration desk to show their decision. (Please see the script for introducing the clinical assessment.)

At the beginning of the consultation, the trainee will introduce the IDCAP clinician to the patients who gave verbal consent. The IDCAP clinician will explain to the patient that two clinicians will be routinely conferring about every patient. The patient should not be alarmed that the discussion could reflect severe or unusual illness. The IDCAP clinician will remind the patient that s/he has a right for the observation to stop at any time.

**Data Storage**

Please note that the IDCAP employees sign a confidentiality agreement as a condition of their employment at IDI.

The case scenarios will be completed at IDI and trainee scores will be reported in the IDI training data base as part of IDI’s monitoring and evaluation practices. Each trainee will be assigned a trainee ID number and s/he will be identified by it in the IDI training data base. The written responses will be stored in a locked file cabinet at the IDCAP office. The records of trainees who agree to share a confidential record will be assigned a second IDCAP number. The record will not contain the trainee’s name or demographic information that would identify him/her. The record will have an IDCAP number, and a confidential key will link the IDCAP number to the IDI training records. The confidential key will be stored securely and only the people listed on the consent form will have access to it.

The clinical observation will be performed by an IDCAP clinician who will spend two days at the trainee’s health facility observing a trainee’s clinical practice with seven patients in the outpatient clinic and seven patients in the HIV/AIDS clinic. The data collected by the IDCAP observer will be used to score the trainee’s clinical performance using a score sheet. The observation data will be identified by the trainee’s ID number in the data base. The IDCAP clinician will record anonymous data on patient history, physical examination, and laboratory investigations and other imaging in order to assess the quality of the trainee’s clinical practice. No identifying information about the patient will be recorded.

Each of the mobile teams who observe the trainees will travel with a small, locked box in which to store the observation score sheet and anonymous patient data when traveling. Written information will be stored in a locked file cabinet at the IDCAP office. The records of trainees who agree to share a confidential record will be assigned same IDCAP number and the data will be managed in the same way as the case scenarios with one exception. For the end-of-intervention assessment of outpatients, the principal investigator will transport the clinical assessment forms to Seattle WA for coding, where they will be stored in a locked file cabinet in her office, and then returned to the IDCAP office for data entry.

**Patient Safety**

If an emergency case is identified by the clinic team or the IDCAP clinician during the clinical observation of the trainee, the IDCAP clinician will stop the observation, support the trainee in the management of the emergency patient, and thereafter complete the Emergency Case Reporting Form.

### 3.2.6 Limitations

The success of the proposed intervention will depend on our ability to integrate it within other existing training activities, to avoid duplication of the considerable efforts to date. The diversity of training providers in Uganda is substantial, and coordination of those efforts must happen from within the Ugandan government. IDI is well positioned to facilitate this effort for IDT, as it has effectively worked with the MOH to have the Joint Uganda Malaria Training Program approved by the National Malaria Control Program and the National Malaria Case Management Technical Working Group. Many organizations have made major contributions to the body of training materials and learning approaches, and we intend to work closely with those partners to ensure our collective success. Most importantly, the WHO has established training materials that all parties view as essential and complementary, and working to ensure that this program builds on that foundation will be a high priority.

Another possible limitation would be attrition of the health professionals who have been trained. In collaboration with the Ministry of Health (MOH), IDCAP has built-in mechanisms to increase retention in the evaluation sites and institutionalize the quality improvements.

1. The district health officers have been asked to refrain from transferring the trainees in the core Infectious Disease Training (IDT) for 12 months and preferably 24 months after attending the IDT.
2. IDT trainees may be motivated to stay at the IDCAP sites until they complete the nine-month IDT, because they will benefit from distance learning and booster sessions at IDI as well as receiving an IDI certificate. In addition, the MOH’s Director of Clinical Services has encouraged IDI to make the IDT a degree program at Makerere University. Although creating a degree program will take longer than nine months, there is a precedent for awarding a degree retroactively. Participants in Hospice Uganda’s nine-month, palliative care course were awarded a degree retroactively when the course became a degree program.
3. Based on the SOMREC’s comments, IDI has decided to offer a fifth session of the IDT mid-way through phase 1. At sites where the IDT trainees have transferred or otherwise left, a replacement will be identified to attend this additional session.
4. Whereas the IDT will target a small number of staff in each facility, the on-site support seeks to institutionalize better clinical skills and quality improvement. The on-site support focuses on health professionals who are actively providing care at the site, which the IDCAP site assessment shows are the people with longer job tenure. When the practices are adopted by the team at the site, they can be shared as part of the orientation of new staff so that the skills and processes are effectively transferred.

# 3.3 Facility Cost and Performance Indicators

### 3.3.1 Methods

The second set of research question are: Does OSS, significantly improve clinic performance and health outcomes? If yes, are the OSS activities cost-effective? To answer these questions, 36 sites will receive the OSS activities for nine months, but 18 will be randomly assigned to receive the OSS in phase 1 and the others will be phase 2 sites that will serve as a control group in phase 1 and receive OSS months later. The unit of analysis will be the site, and the evaluation design is a randomized trial of sites on key performance indicators.

These research questions are reflected in specific objectives 2-5:

1. To evaluate the effectiveness of on-site support services in improving clinic performance
2. Estimate the cost-effectiveness of on-site support services
3. Compare two methods for estimating cost-effectiveness: a) economic modeling in which effectiveness is measured by original data on performance indicators at 36 sites and published estimates of the effect of the performance indicators on Disability Adjusted Life Years (DALYS), and b) estimates in which effectiveness is measured original verbal autopsy data on mortality among children five years or less.

5. Evaluate the effectiveness of bi-monthly OSS for six months on clinical performance at Phase 1 sites after monthly OSS for nine months during Phase 1.

For specific objectives 2 and 5, sites will be evaluated on the basis of a series of laboratory and clinical performance indicators for infectious diseases on the following areas: 1) preventing maternal to child transmission, 2) general HIV Care, 3) ART, 4) TB, 5) malaria, 6) emergency treatment and triage as presented in Table 1.

The laboratory performance indicators will be based on external quality assurance (EQA) data from three sources:

- National Tuberculosis Reference Laboratory data on acid-fast bacilli (AFB) smears for all IDCAP sites from Oct 2009 to present
- Uganda Virus Research Institute (UVRI) dry tube samples for HIV rapid test (3 positive and 3 negative per site) to test HIV panel proficiency
- Label and collect the 1^st^ negative malaria blood smear and last positive smear each day for one month at every site.  Mobile team lab technicians will check the labels against the lab register to confirm they are the 1^st^ negative and last positive.

The clinical performance indicators will be based on Health Management Information System (HMIS) data or field-tested alternatives to the HMIS forms. For example, the Uganda Malaria Surveillance Program (UMSP) patient visit form will replace the medical form 5. In addition to the UMSP forms, data will be extracted from the Antenatal Care Register, Maternity Register, Post Natal Care Register, Inpatient Register, HIV/ART cards, and TB Registers, and the laboratories’ Dried Blood Spot, Polymerase Chain Reaction DBS-PCR register .

The patient visit form (or modified version of it) would be completed by the health facility staff for every patient who visits the outpatient department. The form identifies patients by their name and outpatient department number. For most records, the data base will be anonymous in the sense that the names will not be entered into the data base and the patient records will not be linked across visits. For records that involve follow-up such as a referral or initiation of TB, the data will be confidential in the sense that names will be entered in the data base so that the information can be linked to records at the referral institution or the TB registers.

For the registers and HIV/ART cards, records will be checked for accuracy and completeness and individual data will be extracted at regular intervals. The records will be confidential in the sense that identifying information will be entered in the data base so that records can be linked between registers and cards, and individuals who are lost-to-follow-up can be traced.

All of these data are routinely collected and analyzed by the UMSP at Makerere Unversity or the Ministry of Health’s Quality of Care Initiative (QCI) without undergoing human subjects/ethics review. The IDCAP evaluation is similar to other activities that seek to improve the quality of care to meet existing Ministry of Health guidelines rather than to test new procedures or medical devices.

For specific objective 4, cost-effectiveness will be estimated with two methods. The first method is a model that combines original data on the performance indicators described in Table 1 at 36 sites and the cost of the capacity-building programs with published estimates of the cost of treatment and DALYs saved. The second method is based on original data on mortality of children under five years as described in Section 3.4 below, instead of performance indicators and DALYs. In other words, the denominator of the incremental cost effectiveness ratio will be measured two ways: economic modeling in which effectiveness is measured by original data on performance indicators and published estimates of the effect of the performance indicators on DALYS, and b) estimates in which effectiveness is measured with original verbal autopsy data on mortality.

The first method was proposed by Mason et. al. to estimate “policy-cost-effectiveness.”[[19]](#endnote-20) Their method grew out of observation that many cost-effective treatments were not adopted by providers. For example, the cost-effectiveness of co-trimoxazole prophalaxis is well established. Pitter et. al estimated that providing it to all HIV-infected people is cost-saving ($2.5 per person year) and providing it only to people in WHO clinical stages 2, 3, and 4 cost $22.23 per DALY gained relative to no prophalaxis.[[20]](#endnote-21) Yazdanpanah et.al. estimated that providing it to people in WHO clinical stages 2, 3, and 4 cost $150 per life year saved relative to no prophalaxis.[[21]](#endnote-22) This evaluation does not seek to replicate the cost-effectiveness analysis of this treatment. It evaluates the cost-effectiveness of a capacity-building intervention that promotes the adoption of the treatment at health facilities.

Interventions such as training and CQI are necessary for providers to adopt a treatment, and also involve costs and varying degrees of effectiveness. Mason et. al.’s method combines published estimates of the cost-effectiveness of the treatment with original research on the cost and effectiveness of interventions that encourage providers to adopt them. Please see the example in Table 2 below. Part A simply repeats the Pitter et. al. calculation of the saving in cost for cotrimoxazole prophalaxis for all HIV-infected people cited above. Part B is a hypothetical comparison of the cost-effectiveness of two capacity-building interventions to promote the treatment. The total cost of the capacity-building intervention plus treatment is more than the treatment alone, and the combination is no longer cost saving. The cost of classroom training is lower that classroom training plus OSS, but so is the effectiveness. As shown in this example, the incremental cost-effectiveness ratio relative to no prophalaxsis is calculated and OSS is more cost-effective.

This method explicitly recognized that there are valid reasons why cost-effective treatments are not adopted and builds interventions that promote their adoption onto published estimates. Mason et. al. list several characteristics that improve “policy cost-effectiveness” that apply to this analysis: 1) lower cost of the capacity-building program, 2) longer duration of the effect of the capacity-building program, 3) greater increase in the number of patients who use the treatment, and 4) greater gain in quality adjusted life years or DALYs of the treatment.

Original data on the cost and effectiveness of the capacity-building intervention will be collected. Cost data (e.g. B1 in Table 2) are available from IDI’s cost-accounting system, URC financial records, and district and facility personnel records. Training costs will be analyzed as an investment and amortized over a time period that reflects changes in guidelines and staff turnover (3 to 5 years). The effectiveness of the capacity-building program at changing provider behaviour. (e.g. B3 in Table 2) will be estimated from the performance indicators described above.

| **Table 2. Sample calculations of the cost-effectiveness ratio of capacity-building programs for cotrimoxazole prophalaxis** | | | |
| --- | --- | --- | --- |
|  |  |  |  |
|  | **No prophalaxis** | **Cotrimoxazole prophalaxis for all HIV infected people** | |
| **A. Pitter et.al. estimates** |  |  | |
| A1. Net treatment cost | $49.19 | $46.69 | |
| A2. Number of DALYS lost | 0.13430 | 0.06861 | |
| A3. Incremental benefit, i.e.DALYS gained |  | 0.0657 = 0.13430 - 0.06861 | |
| A4. Incremental cost |  | -$2.5 = $46.69 - $49.19 | |
| A5. Incremental cost-effectiveness ratio |  | Cost saving | |
| **B. Estimates with capacity-building** |  | **Classroom training** | **Classroom training plus OSS** |
| B1. Training cost |  | $10 | $110 |
| B2. Net program cost with training |  | $56.69  = $46.69 + 10 | $156.69  = $46.69 + $110 |
| B3. Effectiveness of training program at changing provider behavior |  | 5% | 90% |
| B4. Incremental benefit, i.e. DALYS gained |  | 0.0033  = 0.0657 * 0.05 | 0.0591  = 0.0657 * 0.9 |
| B5. Incremental cost |  | $7.50 | $107.50 |
| B6. Incremental cost-effectiveness ratio |  | $2273  = $7.50 / 0.0033 | $1819  = $107.5 / 0.0591 |

**Table 3. Examples of published estimates of cost-effectiveness and facility performance**

| **Treatments** | **Published estimates of cost-effectiveness** | **Number of patients treated** |
| --- | --- | --- |
| Preventing Maternal to Child Transmission | Sweat (2004),[[22]](#endnote-23) | Number of pregnant women with HIV who are referred to PMTCT |
| Cotrimoxazole prophalaxis | Pitter (2007), Error: Reference source not found Yazdanpanah (2005)Error: Reference source not found | Number of HIV+ patients in general care who are prescribed daily Cotrimoxazole therapy |
| Anti-retroviral Therapy | Goldie (2006),[[23]](#endnote-24) Badri (2006)[[24]](#endnote-25) | Number of ART naïve HIV+ patients who are eligible for ART who are started on 1st line ART therapy |
| DOTS for Tuberculosis | Baltussen[[25]](#endnote-26) | Number of TB patients detected and cured under directly observed treatment short course (DOTS) |
| Malaria treatment with artemisinin-based combination therapy (ACT) | Morel (2005)[[26]](#endnote-27) | Number of patients with a positive laboratory test for malaria (blood smear) who are treated with ACT |

The original data will be combined with published estimates on the effectiveness of treatments. In Table 3, columns 1 and 2 present examples of treatments that will be featured in the OSS and published estimates of their cost and effectiveness. Column 3 presents data on the number of patients treated that will be collected as indicators of the effectiveness of OSS (i.e. B3 in Table 2).

The cost-effectiveness analysis will be based on WHO’s Choosing Interventions that are Cost-Effective (CHOICE)[[27]](#endnote-28) from a health sector budget perspective.[[28]](#endnote-29) Although the United States Panel on Cost-Effectiveness in Health and Medicine recommended that all cost-effectiveness analyses include a reference case from the social perspective,[[29]](#endnote-30) the most important difference between the health sector budget and social perspectives is the omission of patient and family costs, such as informal home care, patient time when using health services, and transportation costs. Data will not be collected on patient time and transportation costs. Some experts have advised that including informal home care and patient time in cost analysis adds measurement error.[[30]](#endnote-31)

The health sector perspective includes the time of health professionals. Estimates of the cost of the capacity-building intervention will include the opportunity cost of time when health professionals are away from the site at IDI and on-site participating in OSS. District and facility personnel records will be used to identify the profession of participants and the average salary for that profession will be used in the cost estimates.

Original data on the costs of treatments will also be collected, including staff, drugs and laboratory tests. The cost of staff time will be calculated as the opportunity cost of time as described above. The cost of drugs and laboratory tests will include: HIV test kits, cotrimoxazole, first line ART, TB drugs, coartem, reagents for laboratory tests. Data on the quantities of the drugs and reagents reported on stock cards will be multiplied by the MOH unit price.

The published estimates of effectiveness of treatment (e.g. A3 in Table 2) will be measured in life DALYs or life years. These effectiveness measures serve as a common denominator so that costs and effects can be added across treatments. Weaver et. al. used this method to estimate the cost effectiveness of an influenza and pneumococcal vaccination program and found that a combined program was more cost-effective than a program that focused on a single vaccine.[[31]](#endnote-32) This feature is potentially the key to showing that OSS, which might appear expensive for a vertical program, would become cost-effective in the context of an integrated infectious disease capacity-building program.

A cost analysis will be conducted from the providers’ prospective, and focus on workload. Some treatments require spending more time with each patient, which may not be feasible for clinicians who see 80 or more patients per day. The treatment would be more attractive to clinicians if additional staff were trained to provide it, so that the patient load is shared among more staff. It would also be more attractive to clinicians if it reduced the number of visits per episode of illness.

Data on the allocation of time across health facility staff will be available from the revised medical form 5. For each outpatient visit, data are reported on the clinical and laboratory professional who provided care. Data are also reported on whether a patient was a “new attendance” or “re-attendance,” meaning a patient who has visited the health center more than once for the same episode of illness. These data will be used to compare the number of patients treated per clinical and laboratory professional between the intervention and control sites. The analysis will address whether or not the patient load is shared more broadly. In addition, the total number of patients treated will be compared between the intervention and control sites. The number of patients treated will distinguish between new attendance and re-attendance. As in the cost-effectiveness analysis, district and facility personnel records will be used to identify the profession of clinical and laboratory staff and the average salary for that profession will be used in the cost estimates.

### 3.3.2 Design

Hypothesis 2 will be tested with comparisons of the mean difference (effect size) in the performance indicators described above between Phase 1 and Phase 2 sites. The difference will be calculated between baseline and month nine. The primary analysis will be a regression of clinic-level data where the performance indicator after nine months is the dependent variable. The independent variables will include a dummy variable for whether the site was in Phase 1 or Phase 2 sample, and the indicator at baseline.

Other independent variables will include process indicators that describe the health system factors affecting performance at the facility level as shown in Table 1. Although the training program will not focus on these indicators, we will attempt to control for them in our analyses. MOH and URC have developed a set of indicators for HIV/AIDS services that are tracked during their monthly visits that will be extended to other infectious diseases. Examples include availability of HIV test kits and antiretroviral drugs, and presence of working laboratory equipment. Process measures for the laboratory are routine baseline and end-of-intervention assessments of laboratory characteristics and the performance of individual laboratory professionals at all sites. Data on skills of laboratory professionals will be collected as individual assessments as part of the OSS break-out sessions.

URC recently introduced a measure of employee engagement as a CQI process indicator that will also be collected for IDCAP.[[32]](#endnote-33) The measure is a 16-item questionnaire with questions such: “I know what is expected of me at work” and a likert scale with five response categories (e.g. strongly disagree). Staff will be asked to complete an employee engagement questionnaire three times during the on-site support: 1) at the beginning of phase 1, 2) end of phase 1, and end of phase 2. The employee engagement questionnaire will be administered by the data technicians during their quarterly visits all IDCAP sites.

The cost-effectiveness analysis based on the Mason et. al. model will be calculated as demonstrated in Table 2. The analysis will have two types of sensitivity analysis. One-way sensitivity analyses will be used to examine the magnitude of the change in the cost effectiveness ratio from varying specific parameters. For key parameters, such as the effectiveness of the intervention, a *quasi*-confidence interval will be calculated based on the confidence intervals of parameter for the dummy variable for whether or not the site was in Phase 1 or Phase 2.**[[33]](#endnote-34)** The cost of the intervention will not be included, because it will be deterministic. These calculations will also include the cost and effectiveness of treatments based on confidence intervals of those parameters in the literature.

### 3.3.3 Setting

Cost and facility performance data will be collected from all 36 sites. See Section 3.1.3 for a list of the site selection criteria.

### 3.3.4 Population

For the purpose of the sample size calculation, the unit of analysis is the site. Calculations of the number of sites required and statistical power focused on two malaria indicators and site-level data from eight Joint Uganda Malaria Training Program sites 120 days before training and 120 days after training. The mean change and standard deviation among the eight sites are in columns 1 and 2 of Table 4. The proposed evaluation is designed to detect an 18- point (20-point) difference between the treatment and the control group. Calculations of the number of sites required were based on a power of 80% and alpha of .05 using the sample size program in Stata version 10. Table 3 shows four calculations using two indicators for two different age groups. Column 3 indicates that 18 sites are required per treatment group to achieve 80% power for the first indicator for patients 5 years or more, but that the proposed design of 16 per group will be adequate for the others. Similarly, column 4 indicates that the proposed design will have only a 76% chance of detecting a difference of 10 points for the first indicator for patients 5 years or more, but that that there is a high probability (81% to 100%) of detecting differences for the other indicators. It is likely that the proposed design will provide sufficient power for other indicators as well. If site-level covariates are added to the site-level regression analysis, the power may be higher than shown here.

**Table 4: Sample size calculations to test the effect of on-site support on malaria indicators**

| **Indicator** | **Mean difference** | **Standard Devia-tion** | **Numbe of sites required for power of 80%** | **Power of estimates when number of sites is 16** |
| --- | --- | --- | --- | --- |
| **% of patients with a lab test for malaria among people suspected to have malaria** |  |  |  |  |
| Children < 5 years | 18 | 18 | 16 | 76% |
| Patients 5 or more years | 18 | 19 | 18 | 81% |
| **% with a negative laboratory test for malaria who are treated for malaria** |  |  |  |  |
| Children < five years | 20 | 12 | 6 | 100% |
| Patients 5 or more years | 20 | 10 | 4 | 100% |

### A sample size calculation was not performed for the cost of the intervention, because IDCAP is the only entity providing it; i.e. the information is deterministic.3.3.5 Ethical Considerations

**Consent**

a) Sites. The district health officers where each of the sites is located and facility in-charge have been informed about IDCAP during meetings in June and August 2009. At the meetings, the roles and responsibilities of the district, health facility and IDCAP staff in building capacity and collecting data were explained. In addition, the randomization process was explained, including the role of the Phase 2 sites. Potential sites were asked to participate only if they would accept to be Phase 2 sites if they were randomly assigned to that phase.

b) Facility staff. Facility staff who participate in the OSS will also apply to the IDI. Most of the facility staff who do not attend the core IDT will be assessed on the basis of the facility performance indicators for the site. Their consent will not be required to use the facility cost and performance indicators. An exception is the laboratory staff who will be assessed on their performance during the OSS laboratory breakout sessions to see if they have met IDI standards for laboratory training. The laboratory professionals will be asked to give consent for secondary analysis of training program data. When they apply to the IDI training program, the application form will have two parts: a) course application, and b) consent to share the trainees’ data with the IDCAP evaluation. For the consent form, permission is requested to use trainee data to create a confidential record that will be used for the IDCAP evaluation. As stated on the form, “The decision is voluntary. You should feel completely free to choose not to share the data and to withdraw your data from the analysis without giving a reason.”

The OSS will include classroom sessions and clinical mentoring for all the facility staff. Observation of the staff by the mobile teams is one way that the mobile team members identify topics on which to mentor the staff. Staff will also be encouraged to identify areas in which they would like mentoring using innovative methods such as diaries. The observation is confidential; mobile team members are not supervisors and do not report performance issues to the facility in-charge or district health teams. Feedback will be given in confidence and never in the presence of patients or colleagues. It is possible that the mobile team members will witness gross incompetence on the part of one of the staff. Under circumstances when patient safety is at stake, the mobile team members will report to the facility in-charge or district health team.

Individual data will be collected on the employee engagement questionnaire. To link the responses over time, data will be collected confidentially. Employees will be exposed to minimal risk that might arise from the loss of confidentiality of the data. Each employee will be asked to give verbal consent. As explained on the consent form, completing the questionnaire is voluntary and confidential.

To maintain confidentiality, staff will be assigned a code number that will be used only for the employee engagement questionnaire. The data technicians who administer the questionnaires during the site visit will keep a key linking each staff member to his/her code number. The data technician will write the code number on the questionnaire when they give it to the staff member. The key will be kept in a locked container that the data technicians bring with them to the site whenever the questionnaires are not being administered, and a locked file cabinet at the IDCAP office.

c) Patients. IDCAP poses minimal risk to patients, because facility staff will be encouraged to comply with Ministry of Health guidelines. At this time, we would like to request a waiver from obtaining informed consent from the patients, which is recommended for quality improvement programs that have a rigorous evaluation design.[[34]](#endnote-35) IDCAP will protect the confidentiality of these data.

The exception is patients with HIV. The indicators on mortality for HIV patients will be measured from a cohort of patients who come for a visit during the first three months after the OSS begins. They will be exposed to the minimal risk of loss of confidentiality and asked to give verbal consent for IDCAP to follow their health status for 24 months. Contact information that is reported on HIV/ART cards will be collected. These patients will be asked to check with the evaluation clerk at the site each month when they come for a monitoring visit during treatment and report whether they are working and/or performing normal activities. At the end of phase 1 and 2, they will be offered a transportation refund for travel to the clinic for a total of two payments even if they complete or stop treatment. If the patient misses a monthly visit, the evaluation assistant will use the contact information to find out whether the patient has transferred out, stopped treatment, become bed-ridden or died.

**Data Storage**

Please note that the IDCAP employees sign a confidentiality agreement as a condition of their employment at IDI.

The OSS assessment of laboratory professionals will be reported in the IDI training data base as part of IDI’s monitoring and evaluation practices. Each trainee will be assigned a trainee ID number and s/he will be identified by it in the IDI training data base. The records of trainees who agree to share a confidential record will be assigned a second IDCAP number. The record will not contain the trainee’s name or demographic information that would identify him/her. The record will have an IDCAP number, and a confidential key will link the IDCAP number to the IDI training records. The confidential key will be stored securely and only the people listed on the consent form will have access to it.

The patient visit forms will be stored according to HMIS procedures at the health facility. Records for visits that did not involve follow-up will be assigned a visit ID number in the sequence in which they arrive and not have any identifying information about the patient in the data base. Records for visits that involve follow-up (e.g. someone newly diagnosed with tuberculosis) will be assigned an evaluation ID number and the patient will be identified only by that ID number in the data base. The key that links the patient to the ID number will also be kept in a locked file cabinet at the IDCAP office.

Note that the identifying information of patients with visits that involve follow-up will be transferred to data extraction form that will be used to check registers and HIV/ART cards to see if the follow-up activities occurred.

The registers and HIV/ART Cards will be stored at the health facility according to their standard operating procedures. Data including identifying information about patients will be extracted onto paper forms that will be stored and transported to IDCAP office in locked vehicles. Data will be entered and stored in password protected files of a computer at the IDCAP office. These files will be stored on an external hard drive, rather than a server. Patients will be assigned an evaluation ID numbers (not their patient ID numbers) and they will be identified only by that ID number in the data base. The key that links the patient to the ID number will also be kept in a locked file cabinet at the IDCAP office.

For the cohort of HIV patients, all contact information will be maintained in a locked box at the site by the site evaluation clerk. At the end of the evaluation, the information will be transferred to the IDCAP office. No information identifying the participant or his/her family will ever be released to anyone except for the evaluation clerk and the people listed on the consent form. Information about their health status will be recorded in the data base ID number and will exclude contact and demographic information that could be used to identify him/her. A key linking the ID number to contact information will be securely stored by the IDCAP team for five years and then destroyed.

### 3.3.6 Limitations

The success of the proposed intervention will depend on our ability to avoid duplication of the considerable efforts to date. HCI is well positioned to facilitate this effort for OSS, as it has effectively been run through the MOH since its inception. The prevalence of so many other interventions brings another challenge, which is to filter the noise and accurately assess the *incremental* impact of the proposed capacity-building program. Wherever possible, we will control for the effect of complimentary programs in our sites, but accept as a potential limitation (but ultimately, a mutual benefit for all) the fact that progress will be made on several fronts simultaneously toward higher quality healthcare.

The duration of the evaluation is three years. IDCAP focuses on capacity-building rather than supplying commodities. It is anticipated that patients will receive medications for key infectious diseases such as malaria, tuberculosis, and HIV, including anti-retroviral therapy (ART), through the MOH. People will be initiated on ART with doses allocated by the MOH rather than with IDCAP resources. Consequently, it is not anticipated that drug stock-outs at IDCAP sites after the evaluation will be worse than during the evaluation or than other health facilities in Uganda.

It is possible that IDCAP sites will experience fewer drug stock-outs after the evaluation than before. The continuous quality improvement activities of IDCAP seek to improve the processes of care and may result in improved drug procurement processes such as more accurate estimates the amount of drugs needed and more timely orders. To the extent that these improved processes are institutionalized, the supply of drugs would be better at IDCAP sites.

Please note that data on other indicators such as drug supply will continue at half of the Phase 1 sites for at least nine months after the IDT and on-site support stop, so it will be possible to measure the extent to which the improved processes are institutionalized.

# 3.4 Mortality Survey

### 3.4.1 Methods

The second method of the cost-effectiveness analysis will be a compare the incremental cost estimates described above with mortality data. IDCAP has contracted with the Uganda Bureau of Statistics (UBOS) to measure the impact of the IDCAP intervention on mortality rate among children under five (U5) who live within the 5km radius of each of the 36 health facilities where IDCAP is operating countrywide. The mortality rate is the ratio of the number of deaths among children U5 to the total number of children U5 during a 12 month period. The impact will be measured as the “difference in differences,” i.e. the difference in the mortality rate at 18 phase 1 sites (intervention) compared to the difference at 18 phase 2 sites (control), where the difference in the mortality rate is the average baseline mortality rate minus the mortality rate during Phase 1. A comparison of the difference in mortality in phase 1 sites to phase 2 sites will provide an alternative estimate of the incremental benefit (i.e. B3 of Table 2). The incremental cost effectiveness ratio will be estimated as the ratio of the incremental cost (i.e. B4 of Table 2) to the difference-in-difference in mortality between phase 1 and phase 2 sites.

A 3-year recall period is set for this survey i.e. from April 2008 to the present, including 1.5 or 2 years of baseline data (April 2008-March 2010), and 12 or 18 months of data after the intervention began (April 2010 to September 2011).

### 3.4.2 Design

For the second cost-effectiveness analysis, an alternative measure of effectiveness will be years of life saved. Population-level data is the only valid way to directly measure the effect of the intervention on health outcomes.

It is expected that the capacity-building and quality improvement interventions will reduce mortality by improving the emergency triage and treatment of patients and improving the quality of care for infectious diseases. The Uganda Child Verbal Autopsy Study showed that 75% of children who died received care from a government or private hospital or health center.[[35]](#endnote-36) Given that these children had access to care, it may be possible to save their lives by improving the quality of that care.

When the quality of care improves, utilization of a health facility increases.[[36]](#endnote-37) [[37]](#endnote-38) If the quality of care improves at the Phase 1 sites, they are likely to attract more patients including sicker patients. Paradoxically, as the quality improves the mortality of patients at the facility may increase, because the volume of patients and case complexity increases. Population-level data at the Phase 1 sites would reveal that population-level mortality had decreased because more people are seeking care, even if facility-level mortality increased.

UBOS proposed estimating population-level mortality with a two-part sampling method that builds on recent advances in the Demographic Surveillance Systems in which representatives of local council members at the parish level provide information about recent deaths. Although vital records systems may not be reliable in many African countries, there is a policy in Uganda for citizens to report births and deaths to the local council members. The information is more reliable in rural parishes than urban.

In the first part, the local council member directs the interviewers to households where there is a “high index of suspicion” that a death of a child U5 has occurred. To ensure completeness, a listing of households in each enumeration area (EA) would be created from interviews with all households in collaboration with the local council member. The listing includes an identification number and location for each household, whether or not a death occurred within the household in the last 3 years, and if yes, the age of the person at the time of his/her death.

Households will be selected from 2 strata/tiers for household and birth history interviews.

1. 100% of all households that report a child who was less than five years at the time of his/her death died in the last three years. We expect an average of 4.2 deaths per EA (.028 mortality rate * 150 households/EA * 1 child/household) per year, or 12.6 in 3 years.  Some EA will have more households in the numerator and some will have less.
2. A random sample of 30 households. (See section 3.4.4 below). On average 2.5 household with a death will be among the 30 households selected at random to calculate the denominator.  (30 household in sample/150 household in EA = 0.2 probability of selected at random.  12.6 households in numerator * 0.2=2.5 households.)    We would have their information from strata/tier 1, and they would not need to be re-interviewed in stata/tier 2. On average, this would bring the number of new interviews for strata/tier 2 down to 28 instead of 30. Some EA will have more new interviews for strata/tier 2 and some will have less. Conservatively, we should continue to plan on conducting interviews with 30 household in strata/tier 2.

The IDCAP mortality survey differs from DHS, because we are interested in exposure to the IDCAP intervention, i.e. the length of time that people live within 5 km of the IDCAP site. The following information is needed on each member of the household: a) birth date (age), b) link to “natural” mother, i.e. is birth mother included in household listing**,** c) exposure, i.e. number of months living in household or EA **,** d) Vital status i.e. alive or dead, e) if death, date of death and residence on date of death.

### 3.4.3 Setting

The mortality data will be collected from households that live within five kilometres of the IDCAP sites. See Section 3.1.3 for a list of the site selection criteria.

### 3.4.4 Population

Sample size calculations were based on child mortality results from the evaluation of the Integrated Management of Child Illness (IMCI) in Tanzania.[[38]](#endnote-39) During the two years after IMCI was initiated, the mortality rate in the IMCI districts was 24.4 per 1000 person years compared to 28.2 in the comparison districts, i.e. 13% lower. A similar 13% decrease in mortality was associated with IMCI in Bangladesh.[[39]](#endnote-40)

Professor MaryLou Thompson, PhD, Professor of Biostatistics at the University of Washington derived the equations to calculate the power and sample size for a sampling procedure designed with the following 4 features:

- All U5 deaths in an EA during the period of interest are identified
- The total number of households in the EA is known
- A random sample of c households is taken in the EA
- The number of children U5 in the random sample is determined (this sample may, by chance, include households with a death, but, in general, this sample of c households will be *in addition* to the HHs in which there has been a death)

The sample size and power calculations are based on the following four assumptions.

- 150 households per EA
- On average 1 child U5 per household
- U5 mortality per year at baseline : .028
- 13% reduction in this mortality rate

The number of people per household in the 2006 Uganda Demographic and Health Survey (DHS) was five.[[40]](#endnote-41) The percentage of children under five years was 19.2% or approximately one child under five years per household. This is comparable to the Iganga/Mayuge demographic surveillance site in which a sample of 12,000 households provided data on 64,000 individuals or 5.3 people per household. The sample included 12500 (19.5%) children under five years

For 80% power to detect a 13% reduction from a baseline mortality rate of .028 one would need (ignoring clustering):

| **c^+^** | **#EAs per arm** | **Estimated number of U5 children sampled per arm** |
| --- | --- | --- |
| 10 | 283 | 3942 |
| 15 | 256 | 4846 |
| 20 | 242 | 5790 |
| 30 | 229 | 7770 |

+ Number of HHs sampled in an EA to estimate denominator

* This is U5 children sampled for the denominator + deaths

If we include the effect of clustering,[[41]](#endnote-42) with known denominator and assuming 150 households per EA and sampling of 30 households per EA, the sample size requirements increase to about 260 EAs per arm for a mortality rate coefficient of variation =.25 and to 310 per arm for coefficient of variation=.35.

With a sample of 300 EAs per arm, the household listing would collect mortality data from 45,000 household per arm, and household and birth history data from 12,600 household per arm. Assuming equal sample sizes in each arm, about 35000 children per arm would be required to detect a 14.6 difference. These initial calculations would have to be refined to adjust for the interclass correlation (ICC) associated with sampling several children from the same household.

**3.4.5 Ethical considerations**

**Consent**.

Respondents will be asked to provide consent for the census, household interviews and birth history interview of the mortality data collection process. For the household survey, households will be exposed to minimal risk that might arise from the loss of confidentiality of the data. Households will be asked to give verbal consent, which is the procedure UBOS uses for the DHS. This risk will be minimized with careful management and storage of confidential data (see below). UBOS will collect the data and conduct the consent process according to the laws governing its’ activities.

As part of an amended contract with IDI, UBOS would provide anonymous data files to IDCAP which would be the basis for the final analysis. Only UBOS would have access to identifiable information about the respondents. Any queries from IDCAP analysts have would be with reference to the survey ID number, and IDCAP will not have access to the key that links the survey ID number to the respondents.

**Data Storage**

Please note that the IDCAP employees sign a confidentiality agreement as a condition of their employment at IDI.

All households will be assigned a survey ID number. A key will link the survey ID number to the household location. The field teams will store the location information in locked vehicles which will then transfer to UBOS offices for storage according to their confidential procedures. The survey ID number will be written on the paper questionnaires and used in data bases.

Following UBOS procedures, the sample forms included with this proposal have household identification information on them. This information will also be included on the mortality survey forms.

Information from the census about the location of households in which a death has occurred and the random sample of households will be conveyed to interviewers for the household and birth history interviews using the link between the survey ID number and the household location.

### 3.4.6 Limitations

One limitation is the length of time during which mortality will be observed. Some researchers recommend observing mortality over a 2 year period, because of fluctuation in yearly rates. It will only be possible to observe mortality over a maximum of 18 months, because the duration of the on-site support in Phase 1 will be total of 18 months. It will be possible to observe mortality at baseline over two years.

# REFERENCES

1. WHO, UNAIDS, UNICEF. Towards universal access: Scaling-up priority HIV/AIDS interventions in the health sector. (Geneva: World Health Organization, 2007). Available online at: http://www.who.int/hiv/mediacentre/universal_access_progress_report_en.pdfAccessed on May 19, 2007. [↑](#endnote-ref-2)
2. United States Office of the Global AIDS Coordinator. Power of Partnership: The President’s Emergency Plan for AIDS Relief 2008 Annual Report to Congress. Available on line at: http://www.pepfar.gov/documents/organization/100029.pdf Accessed on May 19, 2008. [↑](#endnote-ref-3)
3. Marchal B, de Brouwere V, Kegels G. HIV/AIDS and the health worker crisis: what are the next steps? *Tropical Medicine and International Health*, 2005; 10 (4): 300-304. [↑](#endnote-ref-4)
4. Committee for Evaluation of the President’s Emergency Program for AIDS Relief (PEPfARf) Implementation. PEPfAR Implementation: Progress and Promise. Sepulveda J, Carpenter C, Curran J, Holzemer W, Smits H, Scott K, and Orza M (eds). (Washington DC: National Academies Press, 2007). [↑](#endnote-ref-5)
5. Kober K, Van Damme W. Scaling up access to antiretroviral treatment in southern Africa: who will do the job? Lancet 2004; 364:103–107. [↑](#endnote-ref-6)
6. Lutalo I, Schneider G, Weaver MR, Oyugi J. Kaye R, Lule F, Scheld WM, McAdam K, Sande MA. HIV/AIDS Training Needs Assessment for Clinicians at ART clinics in Uganda. forthcoming Human Resources for Health. [↑](#endnote-ref-7)
7. Cohen J, Kimaiyo S, Nyandiko W, Siika A, Sidle J, Wools-Kaloustian K, Mamlin J, Carter E J. Addressing the educational void during the antiretroviral therapy rollout. *AIDS* 2004; 18 (15): 2105:21-6. [↑](#endnote-ref-8)
8. Harries AD, Shouten EJ, Libamba E. Scaling up antiretroviral treatment in resource-poor settings. *Lancet*, 2006; 367: 1870-1872. [↑](#endnote-ref-9)
9. Gimbel-Sherr S O. Micek MA, Gimbel-Sherr KH, Koepsell T, Hughes P, Thomas KK, Pfeiffer J, and Gloyd SS. Using nurses to identify HAART eligible patients in the Republic of Mozambique: results of a time series analysis. *Human Resources for Health*. 5: 7. Available online at: <http://www.human-resources-health.com/contents/5/1/7. Accessed June 14>, 2007. [↑](#endnote-ref-10)
10. Gimbel-Sherr K^1,2^, Augusto O^4^, Micek M^1,2^, Gimbel-Sherr S^1,2^, Tomo MI^3^, Pfeiffer J^1,2^, Gloyd S^1,2^

    Task shifting to mid-level clinical health providers: an evaluation of quality of ART provided by non-physician clinicians and physicians in Mozambique. Presentation at the Intenational AIDS Society Meeting in Mexico City, August 2008. [↑](#endnote-ref-11)
11. ? vanGriensven J, deNaeyer L, Uwerai J, Asiimwe A, Gazille C, Reid T, Success with antiretroviral treatment for children in Kigali,Rwanda: Experience with health center/nurse-based care

    *BMC Pediatrics* 2008, 8:39 [↑](#endnote-ref-12)
12. ? Stringer JSA, Zulu I, Levy J, Stringer EM, Mwango A, Chi BH, et. al. Rapid scale-up of antiretroviral therapy in primary care sites in Zambia. *JAMA* 2006; 296: 782-793. [↑](#endnote-ref-13)
13. Morris MB, Chapula BT, Chi BH, Mwango A, Chi HF, Mwanza J, Manda H, Bolton C, Pankratz DS, Stringer JSA, Reid SE. Use of task-shifting to rapidly scale-up HIV treatment services:

    experiences from Lusaka, Zambia. *BMC Health Services Research* 2009, 9:5. [↑](#endnote-ref-14)
14. Mathers CD, Bernard C, Moesgaard IK, et al. Global burden of disease in 2002: data sources, methods and results. Geneva: World Health Organization, 2003. (Global programme on evidence for health policy discussion paper No. 54.) [↑](#endnote-ref-15)
15. Davis D, Obrien, Thomson M A, Freemantle N, Wolf F M, Mazmanian P, Taylor-Vaisey A. Impact of formal continuing medical education: do conferences, workshops, rounds, and other traditional continuing education activities change physician behavior or health outcomes. *JAMA,* 1999; 282(9): 867-874. [↑](#endnote-ref-16)
16. O'Brien MA, Oxman AD, Davis DA, Haynes RB, Freemantle N, Harvey EL. Educational outreach visits: effects on professional practice and health care outcomes. *The Cochrane Database of Systematic Reviews* 1997, Issue 4. Art. No.: CD000409. DOI:10.1002/14651858.CD000409. [↑](#endnote-ref-17)
17. Pariyo GW, Gouws E, Bryce J, Burnham G and the Ugandan IMCI Impact Sudy Team. Improving facility-based care for sick children in Uganda is not enough. *Health Policy and Planning*, 2005; 20(Supplement 1): i58-i68. [↑](#endnote-ref-18)
18. Perol D, Boissel J-P, Broussole C, Cetr J-C, Stagnara J, Chauvin F: A simple tool to evoke physician’s real training needs. *Acad Med* 2002, 77(5):407-410. [↑](#endnote-ref-19)
19. Mason J, Freemantle N, Nazareth I, et. al. When is it cost-effective to change the behavior of health professionals. JAMA 2001; 286 (23): 2988-2992. [↑](#endnote-ref-20)
20. Pitter C, Kahn J G, Marseille E, et. Al. Cost-effectiveness of cotrimixazole prophylaxis among persons with HIV in Uganda. JAIDS 2007; 44 (3): 336-e43. [↑](#endnote-ref-21)
21. Yazdanpanah Y, Losina E, Anglaret X et al. Clinical impact and cost-effectiveness of co-trimoxazole prophylaxis in patients with HIV/AIDS in Cote d’Ivoire: a trial-based analysis. AIDS 2005; 19: 1299-1308.

    [↑](#endnote-ref-22)
22. Sweat M D O’Reilly KR, Schmid G P, et. Al. Cost-effectiveness of nevirapine to prevent mother to child HIV transmission in eight African Countries. AIDS 2004; 18: 1661-1671. [↑](#endnote-ref-23)
23. Goldie S J, Yazdanpanah Y, Losina E, et. al. Cost-effectiveness of HIV treatment in resource-poor settings – the case of Cote d’Ivoire. NEJM 2006; 355: 1141-53. [↑](#endnote-ref-24)
24. Badri M, Maartens G, Mandalia S et. al. Cost-effectiveness of highly active antiretroviral therapy in South Africa. PLOS Medicine 2006; 3(1): 48-56. [↑](#endnote-ref-25)
25. Baltussen R, Floyd K, Dye C. Cost-effectiveness analysis of strategies for tuberculosis control in developing countries. BMJ, 2005; 331: 1364-9 [↑](#endnote-ref-26)
26. Morel C M, Lauer J A, Evans D B. Cost-effectiveness analysis of strategies to combat malaria in developing countries. BMJ 2005; 331: 1299-1305. [↑](#endnote-ref-27)
27. Evans D B, Tan-Torres Edejer T, Adam Taghreed, et. al. Methods to assess the costs and health effects of interventions for improving health in developing countries. BMJ 2005; 331: 1137-1140. [↑](#endnote-ref-28)
28. Drummond MF, O’Brien B, Stoddart GL, Torrance, GW. *Methods for the economic evaluation of health care programmes.* (2^nd^ edition).Toronto: Oxford University Press, 1997. [↑](#endnote-ref-29)
29. Gold, MR, Siegel JE, Russell LB, Weinstein MC, eds. *Cost-effectiveness in health and medicine*, 1996*.* New York: Oxford University Press. [↑](#endnote-ref-30)
30. World Health Organization. Generalized Cost-Effeciveness Analysis: A Guide. Geneva: World Health Organization, 2003. Available online at: http://www.who.int/choice/en/ Accessed on April 14, 2008.

    [↑](#endnote-ref-31)
31. Weaver M, Krieger J, Castorina J, et. al. "Cost-Effectiveness Analysis of a Combined Campaign for Influenza and Pneumococcal Vaccines," Archives of Internal Medicine (2001); v. 161 no. 1: 111-120. [↑](#endnote-ref-32)
32. Harter JK, Schmidt FL, Keyes CL. Well-being in the workplace and its relationship to business outcomes: a review of the Gallup Studies. In Keyes CL, Haidt J (eds) Flourishing: the positive person and the good life. Washington D.C.: American Psychological Association, 2002. [↑](#endnote-ref-33)
33. O’Brien BJ, Drummond MF, Labelle RJ, and Willan A. In search of power and significance: issues in the design and analysis of stochastic cost-effectiveness studies in health care. *Medical Care* 1994; 32 (2): 150-63. [↑](#endnote-ref-34)
34. Miller FG, Emanual EJ. Quality-improvement research and informed consent. NEJM 2008; 358(8): 765-7. [↑](#endnote-ref-35)
35. The Uganda Bureau of Statistics (UBOS), Macro International Inc., and MEASURE Evaluation. 2008. *Uganda Child Verbal Autopsy Study 2007*. Calverton, Maryland, USA: UBOS, Macro International Inc., and MEASURE Evaluation. [↑](#endnote-ref-36)
36. Litvak JI, Bodard C. User fees plus quality equals improved access to health care: results of a field experiment in Cameroon. *Social Science and Medicine* 1993; 37 (3): 369-383. [↑](#endnote-ref-37)
37. Diop F, Yazbaeck A, Bitran R. The impact of alternative cost recovery schemes on access and equity in Niger. *Health Policy and Planning* 1995; 10 (3): 223-240. [↑](#endnote-ref-38)
38. Armstrong Shellenberg J R M, Adam T, Mshinda H, et. al. Effectiveness and cost of facility-based Integrated Management of Childhood Illness (IMCI) in Tanzania. Lancet 2004; 364: 1583-1594. [↑](#endnote-ref-39)
39. ? Arifeen SA, Emdadul DM, Akter T, et. al. Effect of the Integrated Management of Childhood Illness strategy on childhood mortality and nutrition in a rural area of Bangladesh: a cluster randomized trial. Lancet 2009; 374: 393-403. [↑](#endnote-ref-40)
40. ? Uganda Bureau of Statistics (UBOS) and Macro International Inc. 2007. *Uganda Demographic and Health Survey 2006.* Calverton, Maryland, USA: UBOS and Macro International Inc. [↑](#endnote-ref-41)
41. Hayes RJ, Bennet S. Simple sample size calculation for cluster-randomized trials. *International Journal of Epidemiology*,1999; 28: 319-326. [↑](#endnote-ref-42)
